# Exome Sequencing in 53 Sporadic Cases of Schizophrenia Identifies 18 Putative Candidate Genes

**DOI:** 10.1371/journal.pone.0112745

**Published:** 2014-11-24

**Authors:** Michel Guipponi, Federico A. Santoni, Vincent Setola, Corinne Gehrig, Maud Rotharmel, Macarena Cuenca, Olivier Guillin, Dimitris Dikeos, Georgios Georgantopoulos, George Papadimitriou, Logos Curtis, Alexandre Méary, Franck Schürhoff, Stéphane Jamain, Dimitri Avramopoulos, Marion Leboyer, Dan Rujescu, Ann Pulver, Dominique Campion, David P. Siderovski, Stylianos E. Antonarakis

**Affiliations:** 1 Department of Genetic Medicine and Development, University of Geneva Medical School and University Hospitals of Geneva, Switzerland; 2 Department of Physiology and Pharmacology, West Virginia University School of Medicine, West Virginia, United States of America; 3 Centre Hospitalier du Rouvray, Sotteville les Rouen et INSERM U1079, France; 4 1st Department of Psychiatry at the Athens University Medical School, Athens, Greece; 5 Department of Mental Health and Psychiatry, Geneva, Switzerland; 6 Inserm U955, Psychiatrie Génétique, Créteil, France; 7 Université Paris Est, Faculté de Médecine, Créteil, France; 8 Assistance Publique-Hôpitaux de Paris, Hôpital A. Chenevier - H. Mondor, Pôle de Psychiatrie, Créteil, France; 9 Fondation Fondamental, Créteil, France; 10 Epidemiology and Genetics Program in Psychiatry, Johns Hopkins School of Medicine, Baltimore, Maryland, United States of America; 11 Department of Psychiatry, University of Halle, Halle, Germany; 12 Institute of Genetics and Genomics in Geneva (iGE3), Geneva, Switzerland; Odense University hospital, Denmark

## Abstract

Schizophrenia (SCZ) is a severe, debilitating mental illness which has a significant genetic component. The identification of genetic factors related to SCZ has been challenging and these factors remain largely unknown. To evaluate the contribution of *de novo* variants (DNVs) to SCZ, we sequenced the exomes of 53 individuals with sporadic SCZ and of their non-affected parents. We identified 49 DNVs, 18 of which were predicted to alter gene function, including 13 damaging missense mutations, 2 conserved splice site mutations, 2 nonsense mutations, and 1 frameshift deletion. The average number of exonic DNV per proband was 0.88, which corresponds to an exonic point mutation rate of 1.7×10^−8^ per nucleotide per generation. The non-synonymous-to-synonymous mutation ratio of 2.06 did not differ from neutral expectations. Overall, this study provides a list of 18 putative candidate genes for sporadic SCZ, and when combined with the results of similar reports, identifies a second proband carrying a non-synonymous DNV in the *RGS12* gene.

## Introduction

Schizophrenia (SCZ) is a severe mental illness that has a population prevalence of 0.4 to 0.8% [1]. Individuals with SCZ experience psychosis, poor social functioning, and cognitive impairments. SCZ is a highly heritable disorder encouraging research into the genetic component of the disorder [2]. Large genome-wide studies have recently suggested that common single nucleotide polymorphisms (SNPs) collectively account for at least 32% of the variance in SCZ liability [3],[4],[5],[6]. Rare copy number variants (CNVs) have also been shown to contribute to SCZ risk [7],[8],[9],[10]. Despite these encouraging advances, many of the genetic components of the disease await identification. Besides inherited variants, new mutations may also contribute to risk.

A sizeable proportion of SCZ patients do not have a family history of the disease and it has been hypothesized that *de novo* variants (DNVs) could account for some of these sporadic cases [11]. Several lines of evidence support this hypothesis. Firstly, despite the markedly reduced reproductive rate among SCZ patients, the prevalence of the disorder has remained constant in the general population (0.4–0.8%) [12]. DNVs, which are not subject to negative selection, provide an explanation for risk alleles remaining frequent in the population. Secondly, it has been shown that DNV load correlates with paternal age [13], which could explain why older males who have accumulated new mutations are more likely than younger males to father schizophrenic children [14]. Thirdly, DNVs tend to be more deleterious to gene function than inherited ones because they have not been subjected to evolutionary selection (with the exception of mutations incompatible with life) and are thus disease-prone [15]. Consistently, individuals with sporadic SCZ carry significantly more *de novo* CNVs than do controls, whereas individuals with familial SCZ do not [16]. However, subsequent studies confirming a higher rate of *de novo* CNVs among individuals with SCZ compared with controls did not reveal a significant difference between individuals with sporadic or familial SCZ [17],[18].

It has become evident that some fraction of disease alleles may occur as *de novo* events. With recent advances in high-throughput sequencing technologies, one can now identify a considerable fraction of *de novo* variation, including single nucleotide variants (SNVs) and small insertions/deletions (indels), thus increasing the proportion of disease risk that can be explained. Five studies have analyzed the contribution of *de novo* variants in sporadic cases of SCZ [19],[20],[21],[22],[23]. Collectively, these studies reported a mutation rate of ∼1.5×10^−8^ mutation per base per generation, which is consistent with neutral expectations. Regarding the burden of protein-altering variants, some discrepancies exist among these studies. Indeed, Xu et al. (2011 & 2012) observed that individuals with SCZ exhibited a significantly higher ratio of non-synonymous-to-synonymous SNVs compared with controls, whereas Fromer et al. (2014) did not find any significant enrichment.

Here, we further explored the contribution of DNVs to the etiology of sporadic SCZ by sequencing the exomes of 53 affected individuals and of their healthy parents.

## Results

### Schizophrenia trios

Fifty-three individuals with sporadic SCZ (39 males and 14 females) and their unaffected parents were recruited. More than half of the patients had a diagnosis of paranoid schizophrenia (n = 31; 58.4%). The remaining patients had a diagnosis of undifferentiated schizophrenia (n = 6, 11.3%), non-organic schizophrenia (n = 5; 9.4%), disorganized schizophrenia (n = 5, 9.4%), schizoaffective disorder (n = 5, 9.4%), or simple schizophrenia (n = 1; 1.9%). The average age at disease onset was 21.03 years. (Text S1 and Table S1).

### Exome sequencing data

Exome sequencing was conducted for 159 individuals. On average, 206 (±78 SD) million reads were produced per sample. Of these, 198 (±76 SD) million mapped to the reference genome (hg19). After the removal of duplicate reads, 150 (±51 SD) million reads remained. Among these reads, 113 (±40 SD) million were on-target. These reads represented an average coverage of at least 8× for 95.01% (±4.8 SD) of the coding portion of the RefSeq genes. On average, 24,401 (±1,407 SD) variants were detected per individual. Table S2 summarizes the exome sequencing results for each individual.

### Identification of damaging *de novo* variants

In our cohort of 53 proband-parent trios, we identified 47 exonic and 2 intronic conserved splice site DNVs, which were not previously reported in any public SNP database (Table 1). The observed rate of *de novo* events in the RefSeq protein-coding exons was 0.88, corresponding to an exonic point mutation rate of 1.7×10^−8^, in accord with previous reports [21],[24],[25],[26],[27]. The distribution of the number of DNVs per trio did not differ from the Poisson distribution (Chi-square goodness of fit test: p = 0.42; Figure S1). Father's age at childbirth had no significant effect on the number of DNVs in the offspring (R^2^ = 0.0113; p = 0.44; Figure S2A). However, when we separated the father according to the median age at childbirth, we observed a trend towards more DNVs in the children of the oldest fathers (0.79 and 1.08 DNVs in the offspring of the youngest (19–29 years old) and the oldest (30–52 years old) fathers, respectively; one-tailed Student's t-Test; p = 0.097; Figure S2B).

**Table 1:** List of the 49 validated *de novo* variants.

Proband_ID	Gene Name	Gene ID	Mutation type	Nucleotide change	AA change	SIFT	PP2	MT
98708	AHDC1	NM_001029882	missense	c.C1459T	p.R487W	0.00	0.99	0.35
BUZ_406	C5orf4	NM_032385	missense	c.G893A	p.G298E	0.00	1.00	1.00
SZP_trio26.P	C9orf172	NM_001080482	missense	c.G2855T	p.C952F	0.00	1.00	0.99
SP-226	DDX20	NM_007204	missense	c.G163C	p.D55H	0.00	1.00	0.99
15LE-p	KDM3B	NM_016604	missense	c.C4216T	p.R1406W	0.00	1.00	1.00
360	LRRC4	NM_022143	missense	c.C327G	p.N109K	0.00	1.00	0.99
12JC-p	PSMC2	NM_002803	missense	c.G597T	p.E199D	0.02	0.55	1.00
98768	QSER1	NM_001076786	missense	c.T4100C	p.I1367T	0.00	0.77	0.07
SP-227	RGS12	NM_198227	missense	c.G161T	p.R702L	0.08	1.00	1.00
SP-240	SLC22A23	NM_015482	missense	c.T734G	p.L245R	0.00	1.00	0.99
6TP-p	TMEM8C	NM_001080483	missense	c.C184T	p.R62C	0.02	1.00	0.02
2142	USP10	NM_005153	missense	c.C2197T	p.R733W	0.00	1.00	0.99
98757	FAM189A2	NM_001127608	missense	c.C478T	p.P160S	0.09	1.00	0.70
2256	CHD5	NM_015557	missense	c.G3943A	p.V1315M	0.07	0.92	0.26
2150	CILP2	NM_153221	missense	c.G1040A	p.R347Q	0.26	0.41	0.02
88536	DOT1L	NM_032482	missense	c.G2509A	p.A837T	0.81	0.00	0.09
SP-245	HPCA	NM_002143	missense	c.G334A	p.G112S	0.13	0.00	1.00
2262	KDELC2	NM_153705	missense	c.A539G	p.K180R	0.32	0.06	0.00
SZP_trio27.P	KIAA0430	NM_001184998	missense	c.A340C	p.M114L	0.12	0.47	0.01
2265	KIF24	NM_194313	missense	c.G865A	p.V289I	0.07	0.46	0.99
98768	KMO	NM_003679	missense	c.G1381T	p.A461S	0.37	0.00	0.00
SZP_trio26.P	MYLPF	NM_013292	missense	c.G244A	p.V82I	0.11	0.01	0.99
400	PITRM1	NM_001242309	missense	c.A2129G	p.K710R	0.46	0.48	0.95
SZP_trio28.P	SHARPIN	NM_030974	missense	c.T427C	p.S143P	0.17	0.99	0.02
15LE-p	SLC22A9	NM_080866	missense	c.T427A	p.S143T	0.18	0.02	0.00
2265	SNX19	NM_014758	missense	c.G2128A	p.E710K	0.22	0.42	0.12
12JC-p	SYNE1	NM_033071	missense	c.A19898T	p.Q6633L	1.00	0.13	0.03
2145	TIGD7	NM_033208	missense	c.G48A	p.M16I	0.07	0.18	0.01
2150	ZNF844	NM_001136501	missense	c.A1559G	p.K520R	1.00	0.01	NA
18 (3) proband	MAP4K4	NM_001242559	nonsense	c.C1795T	p.R599X			
98706	CHRNG	NM_005199	nonsense	c.C511T	p.Q171X			
SP-236	EIF3B	NM_001037283	splice site	c.2029-1G>C				
88185	SETD1A	NM_014712	splice site	c.4582delAG>-				
2148	FN1	NM_002026	frameshift	c.G277_del	p.A93LfsX26			
233	AJUBA	NM_032876	synonymous	c.T852G	p.L284L			
SZP_trio28.P	ALG11	NM_001004127	synonymous	c.C306T	p.T102T			
404	ANKRD44	NM_001195144	synonymous	c.C882T	p.N294N			
2254	ARHGAP11A	NM_014783	synonymous	c.C1746T	p.S582S			
403	ARRB2	NM_001257331	synonymous	c.G483A	p.P161P			
TON_078	CELF5	NM_021938	synonymous	c.C1359T	p.S453S			
SP-227	FOXO1	NM_002015	synonymous	c.G135C	p.S45S			
SP-226	NCKAP5	NM_207363	synonymous	c.C4398G	p.A1466A			
360	NHSL2	NM_001013627	synonymous	c.C2341T	p.L781L			
98428	ORMDL1	NM_001128150	synonymous	c.G300A	p.K100K			
392	OXA1L	NM_005015	synonymous	c.C591T	p.G197G			
403	PRRC2B	NM_013318	synonymous	c.G5880A	p.P1960P			
2142	RALGDS	NM_001042368	synonymous	c.C1446T	p.T482T			
SP-236	TIMP2	NM_003255	synonymous	c.C651T	p.I217I			
SZP_trio26.P	TMEM55B	NM_001100814	synonymous	c.G300A	p.V100V			

SIFT  =  Sorting Intolerant from Tolerant algorithm, PP2 =  Polyphen2, MT  =  Mutation Taster. In silico prediction scores shaded in grey are considered as damaging.

In addition, we observed four instances of variant alleles being present at low frequencies, ranging from 14 to 21% of reads, indicative of somatic mosaicism in blood cells. We found one nonsense mutation (p.K854X) in *DSG3* and three missense mutations: p.S1006F in *NLRP11*, p.G1352D in *LRRC7*, and p.R1177C in *CC2D2A*. These 4 events were confirmed by Sanger sequencing, which showed a similar level of allelic imbalance (Figure S3). As these post-zygotic mutations were not necessarily present in the brain of the patients, we did not analyze them further.

Among the 49 DNVs, we discovered 15 synonymous and 34 protein-altering DNVs, including 29 missense mutations, 2 nonsense mutations, 2 conserved splice-site mutations, and 1 frameshift insertion/deletion. The non-synonymous-to-synonymous mutation ratio of 2.06 was similar to neutral expectations (2.23) [28]. We did not observe a significant increase in the relative rate of loss-of-function-to-missense mutations in our cohort when compared to controls from published data sets [21],[22],[24],[26],[29],[30] (0.17 versus 0.13, respectively; X-squared  = 0.0765, p-value  = 0.78). The transition-to-transversion ratio for coding sequences was 2.61, consistent with neutral expectations [31]. Thirteen out of 29 missense DNVs were classified as damaging by at least 2 of the 3 prediction algorithms used (SIFT, Polyphen2 and Mutation Taster) (Table 1). Among the nonsense mutations, the first creates a stop codon at residue 599 of the MAP4K4 protein, which would result in a truncation mutant lacking the 617 C-terminal amino acids (1273, R599X). The second nonsense mutation is in codon 171 of the CHRNG gene, which would result in a truncation mutant lacking the 376 C-terminal amino acids (517, Q171X). Concerning the splice site mutations, both affect a conserved “AG” dinucleotide of an acceptor site, one within intron 14 of the EIF3B gene and the other within intron 15 of the SETD1A gene; both are predicted to significantly impact normal splicing (Table S3). Finally, the indel variant corresponds to a single-nucleotide deletion in the *FN1* gene, resulting in a frameshift at Ala93 and a premature stop codon after the introduction of 26 amino acids. The *FN1* gene produces multiple protein isoforms, the shortest of which contains 657 amino acids (NM_054034.2). Globally, 36% of the DNVs identified in this study (18 out of 49) were predicted as damaging (missense) or loss-of-function (nonsense, conserved splice site and frameshift variants).

We did not observe genes recurrently mutated in our cohort. However, when data from all available studies (including ours) were combined [19],[20],[21],[22],[23], 21 genes were found to be recurrently mutated with likely damaging DNVs trios (Table S4). Among these 21 genes, 13 were found to carry non-synonymous *de novo* SNVs. We then determined the probability of such *de novo* events occurring in these 13 genes based on each gene-specific mutation rate and the total number of non-synonymous *de novo* SNVs observed across all five datasets (1,020 SCZ trios). The number of DNVs observed in these 13 genes was in agreement with the null expectation (Table 2). As splice-site mutations and indels were not accounted for in the mutability calculation, we did not determine the probability of observing multiple events in genes carrying these types of mutations. This analysis, using data from all available studies, revealed a short list of 21 candidate genes, among which some may be confirmed as true risk genes upon the analysis of additional sporadic cases.

**Table 2:** Probability of occurrence of the observed number of DNVs in genes recurrently hit by non-synonymous de novo SNVs.

Study	Chr.	Mutation	Gene	AA substitution	p-value*
Gulsuner_2013	22	Missense	CACNA1I	p.797T>M	6.41 x 10^−3^
Gulsuner_2013	22	Missense	CACNA1I	p.1311R>H	
Fromer_2014	5	Missense	CD14	p.152V>M	2.01 x 10^−4^
Fromer_2014	5	Missense	CD14	p.27L>M	
Karayiorgou_2012	1	Missense	DPYD	p.539G>R	5.8 x 10^−4^
Karayiorgou_2012	1	Nonsense	DPYD	p.621W>*	
Fromer_2014	X	Missense	HUWE1	p.4237R>C	1.18 x 10^−2^
Fromer_2014	X	Missense	HUWE1	p.326A>G	
Gulsuner_2013	4	Nonsense	KIAA1109	p.2439Q>*	9.3 x 10^−3^
Karayiorgou_2012	4	Missense	KIAA1109	p.4950Y>D	
Fromer_2014	11	Missense	KIF18A	p.188V>I	2.7 x 10^−4^
Fromer_2014	11	Missense	KIF18A	p.20P>L	
Fromer_2014	1	Missense	LPHN2	p.372P>R	9.01 x 10^−4^
Fromer_2014	1	Nonsense	LPHN2	p.803R>*	
Fromer_2014	10	Nonsense	MKI67	p.372R>*	5.01 x 10^−3^
Gulsuner_2013	10	Nonsense	MKI67	p.857K>*	
Fromer_2014	2	Nonsense	NEB	p.639Y>*	3.2 x 10^−2^
Gulsuner_2013	2	Missense	NEB	p.7908T>M	
Fromer_2014	1	Missense	NIPAL3	p.172V>M	1.3 x 10^−4^
Fromer_2014	1	Nonsense	NIPAL3	p.398R>*	
This study	4	Missense	RGS12	p.702R>L	2.06 x 10^−3^
Karayiorgou_2012	4	Missense	RGS12	p.1120P>L	
Fromer_2014	15	Missense	RYR3	p.2205V>M	1.32 x 10^−2^
Fromer_2014	15	Missense	RYR3	p.4730I>T	
Fromer_2014	17	Missense	STAC2	p.3E>K	1.71 x 10^−4^
Karayiorgou_2012	17	Missense	STAC2	p.110L>P	

*Level of statistical significance after Bonferroni correction was set at 0.05/18.000 = 2.70×10^−6^.

(18,000 =  number of RefSeq genes used).

Functional *in silico* analysis of 375 genes affected by protein-altering *de novo* variants (missense predicted as probably damaging by Polyphen, nonsense, conserved splice site (±2) and frameshift Indels) [20],[21],[22],[23] and this study (Table S5) revealed a marginally significant enrichment for genes involved in cellular component morphogenesis (GOTERM_BP_FAT Gene Ontology, Benjamini p-value  = 3.1×10^−2^) and genes expressed in brain tissues (TISSUE Expression, Benjamini p-value  = 5.4×10^−8^).

## Discussion

Similar to other studies [19],[20],[21],[22],[23], we used whole-exome sequencing to estimate the contribution of protein-altering *de novo* mutations to sporadic SCZ and to identify susceptibility genes. Our study reveals that 34% of the sporadic cases analyzed (18 out of 53 cases) carried a predicted damaging *de novo* variant, and our results provide a list of 18 putative candidate genes. The average number of exonic DNV per proband was 0.88, which corresponds to an exonic point mutation rate of 1.7×10^−8^ per nucleotide per generation. The non-synonymous-to-synonymous and loss-of-function-to-missense ratios did not differ from expectations or from those of controls, suggesting no difference in mutational processes in sporadic cases of schizophrenia. In a recent paper, Kong et al. (2012) reported on the importance of father's age on the risk of disease, including SCZ [13]. This group convincingly showed that the number of *de novo* mutations in one offspring can be explained by the father's age at childbirth. We did not observe any positive correlation between these two variables, probably owing to the limited size of our sample and the range of the fathers' ages at childbirth (Figure S2A,B).

Considering all five of the above-mentioned studies, 1,020 SCZ trios have been sequenced, and 21 genes were found mutated in more than one SCZ-affected individual (Table S4). Among these 21 genes, RGS12 was found mutated in this study (Table 2). At this stage, none of these genes can be considered definitive risk genes for SCZ, as the recurrence of DNVs is not significant after genome-wide correction (Table 2). Because the estimated mutation rate for indels is less accurate than for SNVs, we did not calculate the probability of observing such events in *LAMA2*. However, the fact that there are 3 occurrences of probably damaging DNVs in this gene makes it a candidate for SCZ risk. Larger samples size will be required to unequivocally identify true risk variants in specific genes.

Functional *in silico* analysis of 375 genes affected by protein-altering *de novo* variants revealed a significant enrichment in genes expressed in brain tissues and genes involved in cellular component morphogenesis. These preliminary results suggest that genes involved in neuronal morphogenesis could be relevant for the altered neurodevelopmental processes in schizophrenia [32]. Among the 18 candidate genes identified in our study, *RGS12* has emerged as one of the most interesting candidates in this neurodevelopmental context, as RGS12 is known to coordinate the Ras-dependent signals required for promoting and/or maintaining neuronal differentiation [33], a process that is perturbed in schizophrenic patients [32]. The *RGS12* gene encodes a member of the ‘regulator of G protein signaling’ (RGS) gene family [34]. In PC12 cells and primary dorsal root ganglion neurons, RGS12 sustains nerve growth factor (NGF)-driven ERK activity and, therefore, neurite outgrowth by scaffolding a complete MAPK cascade, including the NGF-receptor TrkA, activated H-Ras, B-Raf, MEK2, and ERK proteins [33]. In addition, RGS12 is observed to be among the most down-regulated proteins in sensory-deprived barrel cortex synapses, suggesting that it may participate in the molecular mechanisms of sensory development [35].

## Methods and Materials

### Schizophrenia trios

The schizophrenia trios (case and healthy parents) were collected at 5 different psychiatric hospitals (Text S1). In each of the families selected, the proband had a diagnosis of schizophrenia, schizoaffective disorder or non-organic psychosis based on the Diagnostic and Statistical Manual of Mental Disorders (DSM-IV). Families for which we could not exclude the presence of psychosis, major depression, bipolar disorder, autism, mental retardation, learning and developmental delays, multiple hospitalizations in psychiatric units, or any somatic disorders in first and second-degree relatives were not considered. This research project was approved by the ethics committee of all participating centers (Ethics committee of the University Hospitals of Geneva). All participants or legally authorized representatives provided their written informed consent.

### DNA extraction and exome sequencing

Genomic DNA extracted from blood was used except for sample SP-226-003 (Mother), whose DNA was extracted from lymphoblastoid cell line. Whole genome amplification was performed on DNA from SP-198-003 (Mother) and 16(1)-Father using the REPLI-g Mini Kit (Qiagen). Exome capture was conducted using the SureSelect Human ALL Exon kits (Agilent Technologies). High-throughput sequencing was performed on a HiSeq2000 (Illumina). Fastq files were processed by our “in-house” pipeline running on the Vital-IT (http://www.vital-it.ch) Center for high-performance computing of the Swiss Institute of Bioinformatics (SIB) [36].

### Identification of *de novo* variants

*De novo* variants were identified using VariantMaster [36]. Practically, heterozygous variants detected in the proband with SAMtools and PINDEL quality scores ≥100 and ≥600, respectively were retained for subsequent analysis. These variants were filtered so as to exclude variants with a MAF>0.01 in dbSNP (http://www.ncbi.nlm.nih.gov/SNP/), 1000Genomes [37], and Exome Variant Server (EVS, NHLBI GO Exome Sequencing Project (ESP), Seattle, WA (URL: http://evs.gs.washington.edu/EVS/)), and variants found within segmental duplications. VariantMaster utilizes the raw (BAM) data to robustly estimate the conditional probability of a variant to be present in the parents. All variants classified as *de novo* by VariantMaster, were subsequently visually inspected using the SAMtools text alignment viewer. Finally, these candidate variants were validated using Sanger sequencing on each family member. The validation rate was at 100% for the SNVs. Most PINDEL calls classified as *de novo* were rejected during visual inspection mainly due to miscalling of indels in homopolymer tracts or trinucleotide repeats.

### Statistical analysis

The mutation rate (M) of each of the RefSeq gene was calculated by adding up mutation rates of each nucleotide taking into account the mutation rates at CpG and non-CpG sites [13].

We used a germline mutation rate of (i) 6.18×10^−8^ per base per generation for transition at non-CpG sites, (ii) 1.12×10^−7^ per base per generation for transition at CpG sites, (iii) 3.76×10^−9^ per base per generation for transversion at CpG site and (iv) 9.59×10^−9^ per base per generation for transversion at CpG sites. Accordingly, the probability of finding a single mutation in a gene G was 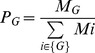 where (G) is the set of all captured genes. Thus, the probability of finding N mutations out of 667 DNVs (number of non-synonymous DNVs observed across this and previously published studies [19],[20],[21],[22],[23] on a specific gene G can be calculated by the binomial distribution: 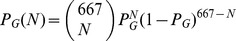. Independently, we may also evaluate the probability of these N mutations in G to be non-synonymous. For each amino acid 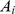, the number of non synonymous mutations can be easily calculated as 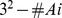 where 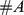 is the number of codons coding for the amino acid 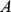. Thus, the probability that a mutation in a gene with coding sequence 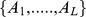 will be non-synonymous is 
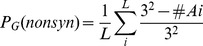
. For each gene, the final P value is calculated as 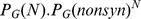.

### Functional In Silico Analysis

Functional annotation of genes carrying protein-altering *de novo* variants was performed by Gene Ontology analysis, using a modified Fisher's exact test with Benjamini correction for multiple testing as implemented in DAVID (http://david.abcc.ncifcrf.gov/).

## Supporting Information

Figure S1 **Rootogram of frequency distribution of** ***de novo*** **events per proband.**
(DOCX)

Figure S2 **A) Age of the father and number of DNVs; B) Average number of** ***de novo*** **variants (DNVs) according to paternal age.**
(DOCX)

Figure S3 **Validated cases of somatic mosaic events.**
(DOCX)

Table S1 **Clinical data for each SCZ trio.**
(DOCX)

Table S2 **Summary of exome sequencing data.**
(DOCX)

Table S3 **Human Splice Finder (HSF) prediction scores for conserved splice site mutations.**
(DOCX)

Table S4 **List of genes hit at least twice by likely damaging DNVs across all five studies (Girard et al. 2011, Xu et al. 20122, 2012, Gulsuner et al. 2012, Fromer et al. 2014, and this study).**
(DOCX)

Table S5 **List of genes carrying de novo protein-altering mutations as reported by Girard et al. (2011), Xu et al. (2012), Gulsuner et al. (2014 and Fromer et al. (2014) and the present study.**
(DOCX)

Text S1 **Detailed sample characteristics.**
(DOCX)
