# Correction: Exome Sequencing in 53 Sporadic Cases of Schizophrenia Identifies 18 Putative Candidate Genes

**DOI:** 10.1371/journal.pone.0141630

**Published:** 2015-10-21

**Authors:** Michel Guipponi, Federico A. Santoni, Vincent Setola, Corinne Gehrig, Maud Rotharmel, Macarena Cuenca, Olivier Guillin, Dimitris Dikeos, Georgios Georgantopoulos, George Papadimitriou, Logos Curtis, Alexandre Méary, Franck Schürhoff, Stéphane Jamain, Dimitri Avramopoulos, Marion Leboyer, Dan Rujescu, Ann Pulver, Dominique Campion, David P. Siderovski, Stylianos E. Antonarakis

## Notice of Republication

Table S1 was published in error. The error was corrected in the HTML and PDF versions of this article on October 12, 2015, and the corrected table is available in the Supporting Information of this article as S1 Table.

## Supporting Information

S1 Table Clinical data for each SCZ trio. (DOCX)
