# Supplementary material for: Exome Sequencing in 53 Sporadic Cases of Schizophrenia Identifies 18 Putative Candidate Genes
Source: PLoS One. 2014 Nov 24;9(11):e112745. doi: 10.1371/journal.pone.0112745 (PMC4242613; doi:10.1371/journal.pone.0112745)

**Figure S1. Rootogram of frequency distribution of de novo events per proband**

The square root of the frequencies are plotted as grey bars and adjusted so that their tops aligned with the Poisson distribution (red line). Distribution of frequencies did not differ from the poisson distribution. Chi-square goodness of fit test returned a pvalue of 0.42


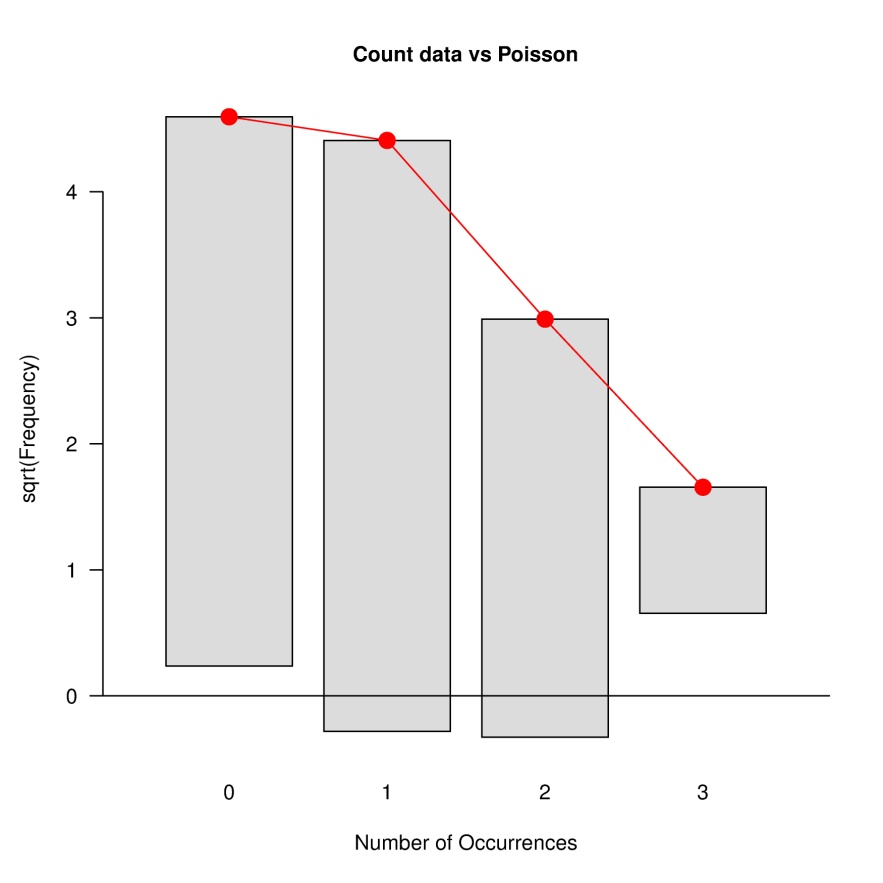

Supplement: Figure S1 — Rootogram of frequency distribution of de novo events per proband. (DOCX) [file pone.0112745.s001.docx]
