# Supplementary material for: Exome Sequencing in 53 Sporadic Cases of Schizophrenia Identifies 18 Putative Candidate Genes
Source: PLoS One. 2014 Nov 24;9(11):e112745. doi: 10.1371/journal.pone.0112745 (PMC4242613; doi:10.1371/journal.pone.0112745)
Supplement: Figure S2 — A) Age of the father and number of DNVs; B) Average number of de novo variants (DNVs) according to paternal age. (DOCX) [file pone.0112745.s002.docx]

**Figure S2**. (A) Correlation between father’s age at childbirth and the number of DNVs in the offspring. (B) Average number of de novo variants (DNVs) according to paternal age. SCZ cases with young fathers (19 to 29 years, which corresponds to the median age) and those with older fathers (30-52 years). The correlation between the number of DNVs in the offspring and the paternal age was tested by Pearson’s correlation

A.


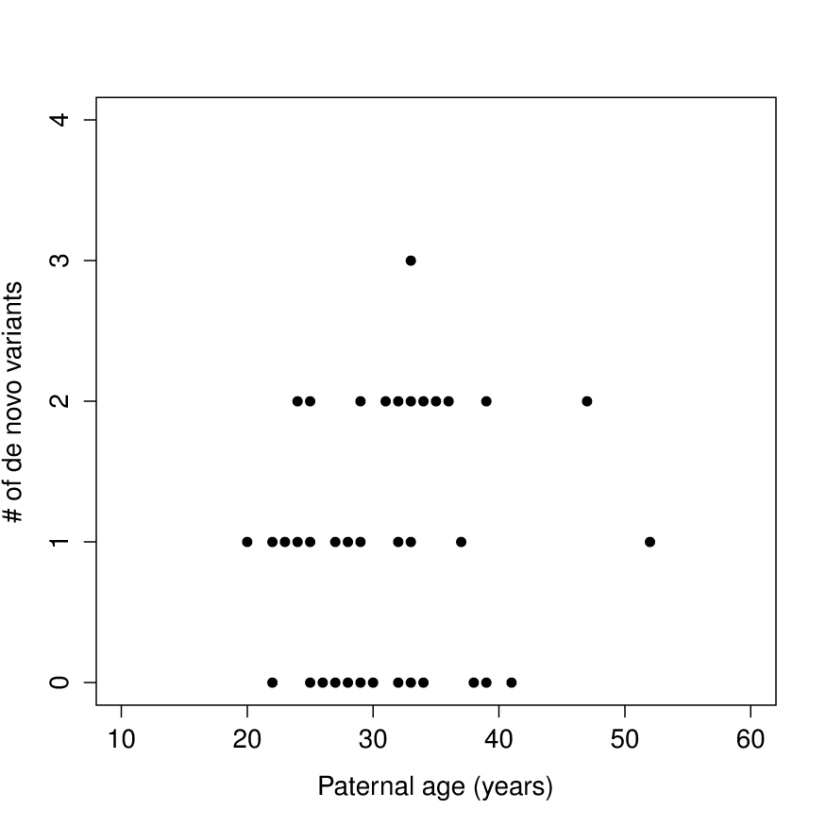


R^2^=0.0113

p=0.44

B.
