# Supplementary material for: Exome Sequencing in 53 Sporadic Cases of Schizophrenia Identifies 18 Putative Candidate Genes
Source: PLoS One. 2014 Nov 24;9(11):e112745. doi: 10.1371/journal.pone.0112745 (PMC4242613; doi:10.1371/journal.pone.0112745)
Supplement: Figure S3 — Validated cases of somatic mosaic events. (DOCX) [file pone.0112745.s003.docx]

**Figure S3: Validated cases of somatic mosaic events**

Sanger sequencing chromatograms of the proband and both parents are presented with the following information: name of the gene, nucleotide and amino acid changes, Samtools DP4 information reporting the number of reads supporting the forward reference allele, reverse reference allele, forward alternative allele and reverse alternative allele, estimates of the percentage of allelic imbalance and proband ID.
