# Supplementary material for: Exome Sequencing in 53 Sporadic Cases of Schizophrenia Identifies 18 Putative Candidate Genes
Source: PLoS One. 2014 Nov 24;9(11):e112745. doi: 10.1371/journal.pone.0112745 (PMC4242613; doi:10.1371/journal.pone.0112745)
Supplement: Table S1 — Clinical data for each SCZ trio. (DOCX) [file pone.0112745.s004.docx]

**Table S1: Clinical data for each SCZ trio**

DOB: Date of birth (DD,MM,YY)

AAC: Age at childbirth (years)

AOO: Age of onset (years)

| **SZP_Trio** | **Fam_ID** | **Gender** | **DOB or AAC** | **AOO** | **Diagnosis** | **Developmental /Learning delays and others** |
| --- | --- | --- | --- | --- | --- | --- |
| **SZP_Trio1** |  |  |  |  |  |  |
| 2142 | Proband | M |  | 19 | Paranoid SCZ | None |
| 2143 | Father |  | 39 |  |  |  |
| 2141 | Mother |  | 26 |  |  |  |
|  |  |  |  |  |  |  |
| **SZP_Trio2** |  |  |  |  |  |  |
| 2145 | Proband | M |  | 18 | Non-organic psychosis | None |
| 2144 | Father |  | 27 |  |  |  |
| 2146 | Mother |  | 29 |  |  |  |
|  |  |  |  |  |  |  |
| **SZP_Trio3** |  |  |  |  |  |  |
| 2148 | Proband | M |  | 21 | Non-organic psychosis | Attention deficit disorder and dyslexia |
| 2149 | Father |  | 52 |  |  |  |
| 2147 | Mother |  | 40 |  |  |  |
|  |  |  |  |  |  |  |
| **SZP_Trio4** |  |  |  |  |  |  |
| 2150 | Proband | M |  | 16 | Paranoid SCZ | None |
| 2151 | Father |  | 32 |  |  |  |
| 2153 | Mother |  | 25 |  |  |  |
|  |  |  |  |  |  |  |
| **SZP_Trio5** |  |  |  |  |  |  |
| 2152 | Proband | F |  | 12 | Paranoid SCZ | Partial epileptic seizure in 2011 |
| 2155 | Father |  | 29 |  |  |  |
| 2154 | Mother |  | 30 |  |  |  |
|  |  |  |  |  |  |  |
|  |  |  |  |  |  |  |
| **SZP_Trio6** |  |  |  |  |  |  |
| *2254* | *Proband* | *F* |  | *16* | *Simple SCZ* | *None* |
| 2255 | Father |  | 33 |  |  |  |
| 2253 | Mother |  | 32 |  |  |  |
|  |  |  |  |  |  |  |
| **SZP_Trio7** |  |  |  |  |  |  |
| 2256 | Proband | F |  | 22 | Non-organic psychosis | None |
| 2257 | Father |  | 23 |  |  |  |
| 2258 | Mother |  | 21 |  |  |  |
|  |  |  |  |  |  |  |
| **SZP_Trio8** |  |  |  |  |  |  |
| 2262 | Proband | M |  | 22 | Non-organic psychosis | None |
| 2263 | Father |  | 20 |  |  |  |
| 2264 | Mother |  | 20 |  |  |  |
|  |  |  |  |  |  |  |
| **SZP_Trio9** |  |  |  |  |  |  |
| 2265 | Proband | M |  | 13 | Non-organic psychosis | Self report of epilepsy in adolescence (not confirmed) |
| 2266 | Father |  | 35 |  |  |  |
| 2267 | Mother |  | 29 |  |  |  |
|  |  |  |  |  |  |  |
| **SZP_Trio10** |  |  |  |  |  |  |
| 233.1 | Proband | M |  | 22 | Paranoid SCZ | Meningitis at the age of 1 year |
| 233.2 | Father |  | 22 |  |  |  |
| 233.3 | Mother |  | 22 |  |  |  |
|  |  |  |  |  |  |  |
| **SZP_Trio11** |  |  |  |  |  |  |
| 73.1 | Proband (M) | M |  | 24 | Paranoid SCZ | None |
| 73.2 | Father |  | 30 |  |  |  |
| 73.3 | Mother |  | 22 |  |  |  |
|  |  |  |  |  |  |  |
| **SZP_Trio12** |  |  |  |  |  |  |
| 266.1 | Proband | M |  | 20 | Disorganized SCZ | None |
| 266.2 | Mother |  | 32 |  |  |  |
| 266.3 | Father |  | 41 |  |  |  |
| **SZP_Trio13** |  |  |  |  |  |  |
| 391.1 | Proband | M |  | 26 | Paranoid SCZ | None |
| 391.2 | Mother |  | 25 |  |  |  |
| 391.3 | Father |  | 27 |  |  |  |
|  |  |  |  |  |  |  |
| **SZP_Trio14** |  |  |  |  |  |  |
| 392.1 | Proband | M |  | 22 | Paranoid SCZ | None |
| 392.2 | Mother |  | 27 |  |  |  |
| 392.3 | Father |  | 33 |  |  |  |
|  |  |  |  |  |  |  |
| **SZP_Trio15** |  |  |  |  |  |  |
| 400.1 | Proband | M |  | 18 | Paranoid SCZ | None |
| 400.2 | Mother |  | 24 |  |  |  |
| 400.3 | Father |  | 25 |  |  |  |
|  |  |  |  |  |  |  |
| **SZP_Trio16** |  |  |  |  |  |  |
| 401.1 | Proband | M |  | 20 | Paranoid SCZ | None |
| 401.2 | Father |  | 28 |  |  |  |
| 401.3 | Mother |  | 26 |  |  |  |
|  |  |  |  |  |  |  |
| **SZP_Trio17** |  |  |  |  |  |  |
| 403.1 | Proband | M |  | 22 | Schizoaffective | None |
| 403.2 | Mother |  | 24 |  |  |  |
| 403.3 | Father |  | 24 |  |  |  |
|  |  |  |  |  |  |  |
| **SZP_Trio18** |  |  |  |  |  |  |
| 404.1 | Proband | M |  | 19 | Paranoid SCZ | None |
| 404.2 | Mother |  | 29 |  |  |  |
| 404.3 | Father |  | 35 |  |  |  |
|  |  |  |  |  |  |  |
| **SZP_Trio19** |  |  |  |  |  |  |
| 360.1 | Proband | F |  | 20 | Disorganized SCZ | Poliomyelitis at the age of 4 years |
| 360.2 | Mother |  | 33 |  |  |  |
| 360.3 | Father |  | 36 |  |  |  |
| **SZP_Trio20** |  |  |  |  |  |  |
| BOU_406_001 | Proband | M |  | 21 | Paranoid SCZ | None |
| BOU_406_002 | Father |  | 29 |  |  |  |
| BOU_406_003 | Mother |  | 28 |  |  |  |
|  |  |  |  |  |  |  |
| **SZP_Trio21** |  |  |  |  |  |  |
| BUZ_408_001 | Proband | F |  | 31 | Schizoaffective | None |
| BUZ_408_002 | Father |  | 32 |  |  |  |
| BUZ_408_003 | Mother |  | 26 |  |  |  |
|  |  |  |  |  |  |  |
| **SZP_Trio22** |  |  |  |  |  |  |
| HAV_405_001 | Proband | M |  | 18 | Paranoid SCZ | Solitary child behavior |
| HAV_405_002 | Father |  | 33 |  |  |  |
| HAV_405_003 | Mother |  | 34 |  |  |  |
|  |  |  |  |  |  |  |
| **SZP_Trio23** |  |  |  |  |  |  |
| LEV_407_001 | Proband | M |  | 27 | Paranoid SCZ | None |
| LEV_407_002 | Father |  | 25 |  |  |  |
| LEV_407_003 | Mother |  | 27 |  |  |  |
|  |  |  |  |  |  |  |
| **SZP_Trio24** |  |  |  |  |  |  |
| TON_078_001 | Proband | M |  | 20 | Paranoid SCZ | None |
| TON_078_002 | Father |  | 25 |  |  |  |
| TON_078_003 | Mother |  | 21 |  |  |  |
|  |  |  |  |  |  |  |
| **SZP_Trio25** |  |  |  |  |  |  |
| TRO_409_001 | Proband | M |  | 21 | Paranoid SCZ | No learning delays |
| TRO_409_002 | Father |  | 27 |  |  |  |
| TRO_409_003 | Mother |  | 22 |  |  |  |
|  |  |  |  |  |  |  |
| **SZP_Trio26** |  |  |  |  |  |  |
| SZP_trio26.P | Proband | M |  | 19 | Paranoid SCZ | Strong decrease in general performance |
| SZP_trio26.M | Mother |  | 29 |  |  |  |
| SZP_trio26.F | Father |  | 33 |  |  |  |
| **SZP_Trio27** |  |  |  |  |  |  |
| SZP_trio27.P | Proband | M |  | 30 | Paranoid SCZ | Strong decrease in general performance |
| SZP_trio27.M | Mother |  | 28 |  |  |  |
| SZP_trio27.F | Father |  | 37 |  |  |  |
|  |  |  |  |  |  |  |
| **SZP_Trio28** |  |  |  |  |  |  |
| SZP_trio28.P | Proband | M |  | 12 | Paranoid SCZ | None |
| SZP_trio28.F | Father |  | 29 |  |  |  |
| SZP_trio28.M | Mother |  | 26 |  |  |  |
|  |  |  |  |  |  |  |
| **SZP_Trio29** |  |  |  |  |  |  |
| SZP_trio29.P | Proband | M |  | 31 | Paranoid SCZ | None |
| SZP_trio29.M | Mother |  | 23 |  |  |  |
| SZP_trio29.F | Father |  | 27 |  |  |  |
|  |  |  |  |  |  |  |
| **SZP_Trio30** |  |  |  |  |  |  |
| SZP_trio30.P | Proband | M |  | 18 | Disorganized SCZ | Slight dysgrammatismus, amnesic aphasia, weak auditive short time memory; reduced, plump, inflexible motor skills |
|  |  |  |  |  |  |  |
| SZP_trio30.M | Mother |  | 35 |  |  |  |
| SZP_trio30.F | Father |  | 41 |  |  |  |
|  |  |  |  |  |  |  |
| **SZP_Trio31** |  |  |  |  |  |  |
| SZP_trio31.P | Proband | M |  | 28 | Paranoid SCZ | None |
| SZP_trio31.M | Mother |  | 29 |  |  |  |
| SZP_trio31.F | Father |  | 39 |  |  |  |
|  |  |  |  |  |  |  |
| **SZP_Trio32** |  |  |  |  |  |  |
| SP-197-001 | Proband | M |  | 18 | Paranoid SCZ | None |
| SP-197-002 | Father |  | 32 |  |  |  |
| SP-197-003 | Mother |  | 32 |  |  |  |
|  |  |  |  |  |  |  |
|  |  |  |  |  |  |  |
|  |  |  |  |  |  |  |
|  |  |  |  |  |  |  |
| **SZP_Trio33** |  |  |  |  |  |  |
| SP-198-001 | Proband | M |  | 20 | Paranoid SCZ | None |
| SP-198-002 | Father |  | 22 |  |  |  |
| SP-198-003 | Mother |  | 21 |  |  |  |
|  |  |  |  |  |  |  |
| **SZP_Trio34** |  |  |  |  |  |  |
| SP-226-001 | Proband | M |  | 27 | Paranoid SCZ | None |
| SP-226-002 | Father |  | 29 |  |  |  |
| SP-226-003 | Mother |  | 32 |  |  |  |
|  |  |  |  |  |  |  |
| **SZP_Trio35** |  |  |  |  |  |  |
| SP-227-001 | Proband | F |  | 25 | Paranoid SCZ | None |
| SP-227-002 | Father |  | 27 |  |  |  |
| SP-227-003 | Mother |  | 22 |  |  |  |
|  |  |  |  |  |  |  |
| **SZP_Trio36** |  |  |  |  |  |  |
| SP-234-001 | Proband | F |  | 26 | Undifferentiated SCZ | None |
| SP-234-002 | Father |  | 27 |  |  |  |
| SP-234-003 | Mother |  | 26 |  |  |  |
|  |  |  |  |  |  |  |
| **SZP_Trio37** |  |  |  |  |  |  |
| SP-236-001 | Proband | M |  | 20 | Paranoid SCZ | No information |
| SP-236-002 | Father |  | 31 |  |  |  |
| SP-236-003 | Mother |  | 30 |  |  |  |
|  |  |  |  |  |  |  |
| **SZP_Trio38** |  |  |  |  |  |  |
| SP-240-001 | Proband | F |  | 26 | Paranoid SCZ | None |
| SP-240-002 | Father |  | 27 |  |  |  |
| SP-240-003 | Mother |  | 25 |  |  |  |
|  |  |  |  |  |  |  |
| **SZP_Trio39** |  |  |  |  |  |  |
| SP-245-001 | Proband | M |  | 19 | Paranoid SCZ | None |
| SP-245-002 | Father |  | 29 |  |  |  |
| SP-245-003 | Mother |  | 30 |  |  |  |
| **SZP_Trio40** |  |  |  |  |  |  |
| 3GF-p | Proband |  |  | 19 | Paranoid SCZ | None |
| 1GF-f | Father |  | 38 |  |  |  |
| 2GF-m | Mother |  | 35 |  |  |  |
|  |  |  |  |  |  |  |
| **SZP_Trio41** |  |  |  |  |  |  |
| 6TP-p | Proband |  |  | 22 | Paranoid SCZ | None |
| 4TP-f | Father |  | 27 |  |  |  |
| 5TP-m | Mother |  | 27 |  |  |  |
|  |  |  |  |  |  |  |
| **SZP_Trio42** |  |  |  |  |  |  |
| 9GP-p | Proband |  |  | 24 | Disorganized SCZ | None |
| 7GP-f | Father |  | 34 |  |  |  |
| 8GP-m | Mother |  | 23 |  |  |  |
|  |  |  |  |  |  |  |
| **SZP_Trio43** |  |  |  |  |  |  |
| 12JC-p | Proband |  |  | 23 | Paranois SCZ | None |
| 10JC-f | Father |  | 34 |  |  |  |
| 11JC-m | Mother |  | 25 |  |  |  |
|  |  |  |  |  |  |  |
| **SZP_Trio44** |  |  |  |  |  |  |
| 15LE-p | Proband |  |  | 18 | Paranoid SCZ | None |
| 13LE-f | Father |  | 47 |  |  |  |
| 14LE-m | Mother |  | 44 |  |  |  |
|  |  |  |  |  |  |  |
| **SZP_Trio45** |  |  |  |  |  |  |
| 18 (3) Patient | Proband |  |  | 20 | Disorganized SCZ | None |
| 16 (1) Father | Father |  | 29 |  |  |  |
| 17 (2) Mother | Mother |  | 25 |  |  |  |
|  |  |  |  |  |  |  |
| **SZP_Trio46** |  |  |  |  |  |  |
| 88060_1001 | Proband | M |  | 28 | Schizoaffective | Developmental delays |
| 88060_2046 | Father |  | 26 |  |  |  |
| 88060_2096 | Mother |  | 21 |  |  |  |
| **SZP_Trio47** |  |  |  |  |  |  |
| 88185_1001 | Proband | M |  | 16 | Undifferentiated SCZ | Developmental delays |
| 88185_2046 | Father |  | 25 |  |  |  |
| 88185_2096 | Mother |  | 25 |  |  |  |
|  |  |  |  |  |  |  |
| **SZP_Trio48** |  |  |  |  |  |  |
| 88536_1001 | Proband | M |  | 23 | Undifferentiated SCZ | None |
| 88536_2046 | Father |  | 28 |  |  |  |
| 88536_2096 | Mother |  | 24 |  |  |  |
|  |  |  |  |  |  |  |
| **SZP_Trio49** |  |  |  |  |  |  |
| 98428_1002 | Proband | M |  | 20 | Undifferentiated SCZ | None |
| 98428_2046 | Father |  | 24 |  |  |  |
| 98428_2096 | Mother |  | 21 |  |  |  |
|  |  |  |  |  |  |  |
| **SZP_Trio50** |  |  |  |  |  |  |
| 98706_1002 | Proband | M |  | 23 | Schizoaffective | Developmental delays |
| 98706_2046 | Father |  | 27 |  |  |  |
| 98706_2096 | Mother |  | 25 |  |  |  |
|  |  |  |  |  |  |  |
| **SZP_Trio51** |  |  |  |  |  |  |
| 98708_1032 | Proband | F |  | 19 | Schizoaffective | None |
| 98708_2046 | Father |  | 33 |  |  |  |
| 98708_2096 | Mother |  | 25 |  |  |  |
|  |  |  |  |  |  |  |
| **SZP_Trio52** |  |  |  |  |  |  |
| 98757_1001 | Proband | M |  | 18 | Undifferentiated SCZ | None |
| 98757_2046 | Father |  | 37 |  |  |  |
| 98757_2096 | Mother |  | 32 |  |  |  |
|  |  |  |  |  |  |  |
| **SZP_Trio53** |  |  |  |  |  |  |
| 98768_1002 | Proband | M |  | 15 | Undifferentiated SCZ | None |
| 98768_2046 | Father |  | 33 |  |  |  |
| 98768_2096 | Mother |  | 30 |  |  |  |
