# Supplementary material for: Exome Sequencing in 53 Sporadic Cases of Schizophrenia Identifies 18 Putative Candidate Genes
Source: PLoS One. 2014 Nov 24;9(11):e112745. doi: 10.1371/journal.pone.0112745 (PMC4242613; doi:10.1371/journal.pone.0112745)
Supplement: Table S2 — Summary of exome sequencing data. (DOCX) [file pone.0112745.s005.docx]

**Table S2: Summary of exome sequencing data**

Age: Age of the parents at childbirth

Agilent: Version of the Agilent SureSelect Human All Exon kit used

Illumina: Samples were sequenced using a Genome Analyzer IIx or a HiSeq2000 using a paired-end 75-nucleotide or 98-nucleotide protocol, respectively.

Total reads: Total number of reads generated for each sample

Properly paired: Total number of reads properly paired

%*: percentage of the total number of reads that were properly paired

rmdup: Number of properly paired reads that remained after removal of duplicates clusters based on identical external coordinates

%**: percentage of properly reads that remained after removal of duplicates clusters

On target: Number of reads that were on target (reads overlapping with the coding part of the RefSeq genes)

% 8x: Percentage of the coding part of the RefSeq genes that were covered at least 8 times

Exonic: Number of exonic variants that were identified for each sample

| **Trios** | **DNA_ID** | **Fam_ID** | **Agilent** | **Illumina** | **Total reads** | **Properly paired** | **%*** | **Rmdup** | **%**** | **On target** | **%***** | **% 8x** | **Exonic** |
| --- | --- | --- | --- | --- | --- | --- | --- | --- | --- | --- | --- | --- | --- |
| **1** | 2142 | Proband | V3 | GAIIx | 160'830'694 | 154'897'828 | 96.31 | 146'988'653 | 94.90 | 96'598'150 | 65.71 | 89.07 | 24'480 |
|  | 2143 | Father | V3 | GAIIx | 76'888'162 | 74'458'856 | 96.84 | 71'585'139 | 96.14 | 46'779'120 | 65.35 | 80.69 | 22'602 |
|  | 2141 | Mother | V3 | GAIIx | 169'695'610 | 132'058'174 | 77.82 | 159'218'912 | 120.57 | 117'701'737 | 73.92 | 87.22 | 22'576 |
| **2** | 2145 | Proband | V3 | GAIIx | 159'752'712 | 152'092'554 | 95.20 | 145'140'691 | 95.43 | 97'951'906 | 67.49 | 87.92 | 24'520 |
|  | 2144 | Father | V3 | GAIIx | 81'298'094 | 77'940'438 | 95.87 | 77'153'065 | 98.98 | 50'827'670 | 65.88 | 81.72 | 21'964 |
|  | 2146 | Mother | V3 | GAIIx | 81'259'950 | 78'641'340 | 96.78 | 76'644'486 | 97.46 | 50'293'492 | 65.62 | 82.55 | 23'084 |
| **3** | 2148 | Proband | V3 | GAIIx | 216'492'494 | 202'778'074 | 93.67 | 185'023'945 | 91.24 | 120'989'624 | 65.40 | 90.33 | 25'671 |
|  | 2149 | Father | V3 | GAIIx | 72'759'490 | 70'644'224 | 97.09 | 68'760'366 | 97.33 | 46'112'366 | 67.06 | 81.55 | 22'824 |
|  | 2147 | Mother | V3 | GAIIx | 72'567'154 | 70'200'044 | 96.74 | 68'930'241 | 98.19 | 44'914'723 | 65.16 | 80.60 | 23'735 |
| **4** | 2150 | Proband | V3 | HiSeq | 106'558'070 | 100'532'432 | 94.35 | 88'276'344 | 87.81 | 65'109'558 | 73.76 | 88.90 | 26'454 |
|  | 2151 | Father | V3 | HiSeq | 104'254'458 | 98'324'726 | 95.27 | 85'077'952 | 85.66 | 62'045'988 | 72.93 | 88.44 | 22'549 |
|  | 2153 | Mother | V3 | HiSeq | 98'312'264 | 93'574'560 | 94.22 | 85'479'111 | 91.35 | 56'053'146 | 65.58 | 87.64 | 27'895 |
| **5** | 2152 | Proband | V3 | HiSeq | 120'468'796 | 111'455'072 | 92.52 | 107'335'474 | 96.30 | 73'394'440 | 68.38 | 89.84 | 22'752 |
|  | 2155 | Father | V3 | HiSeq | 128'645'584 | 120'130'598 | 93.38 | 114'029'200 | 94.92 | 77'921'545 | 68.33 | 90.40 | 22'664 |
|  | 2154 | Mother | V3 | HiSeq | 115'302'578 | 107'274'938 | 93.04 | 98'500'842 | 92.75 | 67'561'480 | 67.90 | 89.18 | 22'606 |
| **6** | 2254 | Proband | V3 | HiSeq | 98'359'360 | 91'859'028 | 93.39 | 85'190'101 | 92.74 | 61'130'877 | 71.76 | 87.98 | 22'585 |
|  | 2255 | Father | V3 | HiSeq | 108'482'598 | 102'254'048 | 94.26 | 95'085'344 | 92.98 | 68'238'671 | 71.77 | 88.95 | 22'873 |
|  | 2253 | Mother | V3 | HiSeq | 125'973'346 | 118'345'266 | 93.94 | 111'075'594 | 93.86 | 79'680'891 | 71.74 | 90.00 | 23'150 |
| **7** | 2256 | Proband | V3 | HiSeq | 144'626'702 | 136'408'820 | 94.32 | 112'682'014 | 82.61 | 83'978'237 | 74.53 | 90.47 | 22'796 |
|  | 2257 | Father | V3 | HiSeq | 86'012'686 | 80'284'710 | 93.34 | 73'928'192 | 92.08 | 54'854'591 | 74.20 | 87.06 | 21'708 |
|  | 2258 | Mother | V3 | HiSeq | 81'198'962 | 76'141'130 | 93.77 | 66'211'393 | 86.96 | 49'026'492 | 74.05 | 86.10 | 22'228 |
| **8** | 2262 | Proband | V3 | HiSeq | 166'355'014 | 153'920'408 | 92.53 | 134'955'605 | 87.68 | 86'121'354 | 63.81 | 90.37 | 23'409 |
|  | 2263 | Father | V3 | HiSeq | 247'365'424 | 226'590'850 | 91.60 | 168'059'409 | 74.17 | 126'895'125 | 75.51 | 92.90 | 23'782 |
|  | 2264 | Mother | V3 | HiSeq | 284'952'118 | 269'574'240 | 94.60 | 194'143'495 | 72.02 | 143'339'536 | 73.83 | 93.31 | 24'301 |
| **9** | 2265 | Proband | V4 | HiSeq | 231'749'796 | 223'085'540 | 96.26 | 181'716'692 | 81.46 | 142'844'543 | 78.61 | 98.74 | 23'813 |
|  | 2266 | Father | V4 | HiSeq | 235'224'678 | 227'908'228 | 96.89 | 184'455'167 | 80.93 | 141'754'761 | 76.85 | 98.74 | 23'984 |
|  | 2267 | Mother | V4 | HiSeq | 198'617'758 | 187'878'190 | 94.59 | 144'831'159 | 77.09 | 110'982'726 | 76.64 | 98.63 | 23'485 |
| **10** | 233.1 | Proband | V3 | HiSeq | 175'066'810 | 165'943'210 | 94.79 | 115'628'761 | 69.68 | 86'737'845 | 75.01 | 90.96 | 21'945 |
|  | 233.2 | Father | V3 | HiSeq | 104'226'422 | 98'038'872 | 95.02 | 85'910'536 | 86.74 | 65'100'630 | 75.78 | 88.52 | 22'538 |
|  | 233.3 | Mother | V3 | HiSeq | 82'330'214 | 77'524'790 | 94.16 | 65'700'736 | 84.75 | 49'281'301 | 75.01 | 86.09 | 21'710 |
| **11** | 73.1 | Proband | V3 | HiSeq | 146'618'380 | 139'320'066 | 95.02 | 110'020'869 | 78.97 | 82'791'944 | 75.25 | 90.33 | 22'739 |
|  | 73.2 | Father | V3 | HiSeq | 251'630'046 | 237'058'460 | 94.21 | 144'828'402 | 61.09 | 109'207'680 | 75.40 | 92.03 | 23'934 |
|  | 73.3 | Mother | V3 | HiSeq | 124'588'020 | 116'928'206 | 93.85 | 100'464'503 | 85.92 | 75'162'024 | 74.81 | 89.96 | 22'747 |
| **12** | 266.1 | Proband | V3 | HiSeq | 75'559'008 | 71'555'186 | 94.70 | 56'939'715 | 79.57 | 42'591'323 | 74.80 | 84.75 | 22'108 |
|  | 266.2 | Mother | V3 | HiSeq | 263'033'410 | 252'050'758 | 95.82 | 155'001'295 | 61.50 | 117'102'053 | 75.55 | 92.47 | 24'331 |
|  | 266.3 | Father | V3 | HiSeq | 86'469'500 | 81'122'778 | 93.82 | 69'541'539 | 85.72 | 51'473'359 | 74.02 | 86.61 | 22'207 |
| **13** | 391.1 | Proband | V3 | HiSeq | 269'373'500 | 257'004'192 | 95.41 | 173'165'788 | 67.38 | 129'486'202 | 74.78 | 93.07 | 24'131 |
|  | 391.2 | Mother | V3 | HiSeq | 83'295'174 | 78'689'968 | 94.47 | 65'037'983 | 82.65 | 48'482'012 | 74.54 | 86.08 | 21'820 |
|  | 391.3 | Father | V3 | HiSeq | 310'833'314 | 293'576'002 | 94.45 | 205'336'080 | 69.94 | 143'946'711 | 70.10 | 93.92 | 24'511 |
| **14** | 392.1 | Proband | V3 | HiSeq | 307'485'138 | 294'354'522 | 95.73 | 174'552'131 | 59.30 | 129'557'921 | 74.22 | 93.20 | 24'319 |
|  | 392.2 | Mother | V3 | HiSeq | 96'196'770 | 89'687'732 | 93.23 | 77'731'443 | 86.67 | 57'740'866 | 74.28 | 87.77 | 22'439 |
|  | 392.3 | Father | V3 | HiSeq | 93'744'642 | 88'985'082 | 94.93 | 77'680'747 | 87.29 | 58'505'133 | 75.31 | 87.71 | 22'233 |
| **15** | 400.1 | Proband | V3 | HiSeq | 184'424'394 | 173'093'640 | 93.86 | 134'808'537 | 77.88 | 101'161'195 | 75.04 | 91.73 | 23'187 |
|  | 400.2 | Mother | V3 | HiSeq | 123'630'522 | 114'712'724 | 92.79 | 103'480'832 | 90.21 | 77'242'446 | 74.64 | 89.95 | 22'648 |
|  | 400.3 | Father | V3 | HiSeq | 102'731'844 | 96'612'294 | 94.04 | 87'442'815 | 90.51 | 64'681'758 | 73.97 | 88.53 | 21'857 |
| **16** | 401.1 | Proband | V3 | HiSeq | 209'238'758 | 197'066'322 | 94.18 | 128'259'744 | 65.08 | 94'695'610 | 73.83 | 91.07 | 23'386 |
|  | 401.2 | Father | V3 | HiSeq | 270'120'020 | 253'465'330 | 93.83 | 190'183'125 | 75.03 | 141'869'967 | 74.60 | 93.47 | 24'244 |
|  | 401.3 | Mother | V3 | HiSeq | 245'016'244 | 226'765'986 | 92.55 | 181'173'661 | 79.89 | 132'791'023 | 73.29 | 93.08 | 24'300 |
| **17** | 403.1 | Proband | V3 | HiSeq | 161'434'010 | 150'483'668 | 93.22 | 116'300'849 | 77.28 | 86'089'977 | 74.02 | 90.63 | 23'389 |
|  | 403.2 | Mother | V3 | HiSeq | 113'212'542 | 106'413'298 | 93.98 | 90'118'842 | 84.69 | 67'372'317 | 74.76 | 88.78 | 22'736 |
|  | 403.3 | Father | V3 | HiSeq | 109'635'260 | 102'412'354 | 93.41 | 87'713'112 | 85.65 | 65'350'005 | 74.50 | 88.79 | 22'553 |
| **18** | 404.1 | Proband | V3 | HiSeq | 174'540'634 | 164'911'348 | 94.48 | 129'714'580 | 78.66 | 97'110'019 | 74.86 | 91.55 | 23'298 |
|  | 404.2 | Mother | V3 | HiSeq | 234'983'982 | 220'460'170 | 93.82 | 143'744'679 | 65.20 | 107'604'805 | 74.86 | 91.72 | 24'030 |
|  | 404.3 | Father | V3 | HiSeq | 88'005'486 | 82'455'702 | 93.69 | 73'050'666 | 88.59 | 54'292'524 | 74.32 | 87.02 | 21'935 |
| **19** | 360.1 | Proband | V3 | HiSeq | 181'548'182 | 171'467'068 | 94.45 | 126'140'739 | 73.57 | 94'616'797 | 75.01 | 91.15 | 23'690 |
|  | 360.2 | Mother | V3 | HiSeq | 108'682'354 | 102'037'372 | 93.89 | 90'880'572 | 89.07 | 67'338'976 | 74.10 | 88.76 | 22'667 |
|  | 360.3 | Father | V3 | HiSeq | 107'956'460 | 101'041'540 | 93.59 | 92'677'314 | 91.72 | 63'759'470 | 68.80 | 88.42 | 24'131 |
| **20** | BOU_406_001 | Proband | V4 | HiSeq | 253'186'838 | 243'082'086 | 96.01 | 176'781'087 | 72.72 | 132'423'909 | 74.91 | 98.68 | 24'645 |
|  | BOU_406_002 | Mother | V4 | HiSeq | 263'353'280 | 253'108'298 | 96.11 | 189'015'929 | 74.68 | 142'577'101 | 75.43 | 98.75 | 24'596 |
|  | BOU_406_003 | Father | V4 | HiSeq | 245'626'022 | 235'975'420 | 96.07 | 182'151'066 | 77.19 | 138'981'369 | 76.31 | 98.57 | 24'859 |
| **21** | BUZ_408_001 | Proband | V4 | HiSeq | 268'524'644 | 258'939'930 | 96.43 | 181'771'350 | 70.20 | 137'711'522 | 75.76 | 98.61 | 24'864 |
|  | BUZ_408_002 | Mother | V4 | HiSeq | 239'430'032 | 232'929'246 | 97.28 | 198'639'635 | 85.71 | 150'723'371 | 75.50 | 98.74 | 25'355 |
|  | BUZ_408_003 | Father | V4 | HiSeq | 231'202'088 | 225'036'364 | 97.33 | 151'758'146 | 67.44 | 117'235'569 | 77.25 | 98.54 | 24'463 |
| **22** | HAV_405_001 | Proband | V4 | HiSeq | 231'779'400 | 224'302'568 | 96.77 | 178'072'012 | 79.39 | 137'409'707 | 77.17 | 98.68 | 24'779 |
|  | HAV_405_002 | Mother | V4 | HiSeq | 263'566'462 | 255'193'584 | 96.82 | 176'735'298 | 69.26 | 135'720'413 | 76.79 | 98.67 | 25'043 |
|  | HAV_405_003 | Father | V4 | HiSeq | 258'883'204 | 251'195'182 | 97.03 | 201'472'378 | 80.21 | 154'638'800 | 76.75 | 98.65 | 25'218 |
| **23** | LEV_407_001 | Proband | V4 | HiSeq | 262'715'484 | 251'923'290 | 95.89 | 209'772'600 | 83.27 | 159'463'229 | 76.02 | 98.66 | 24'943 |
|  | LEV_407_002 | Mother | V4 | HiSeq | 242'814'180 | 233'920'112 | 96.34 | 193'091'376 | 82.55 | 147'551'663 | 76.42 | 98.66 | 24'939 |
|  | LEV_407_003 | Father | V4 | HiSeq | 256'301'812 | 246'873'546 | 96.32 | 181'694'041 | 73.60 | 138'290'428 | 76.11 | 98.62 | 24'982 |
| **24** | TON_078_001 | Proband | V4 | HiSeq | 255'530'626 | 246'462'146 | 96.45 | 101'495'291 | 41.18 | 74'411'255 | 73.31 | 98.66 | 24'069 |
|  | TON_078_002 | Mother | V4 | HiSeq | 208'912'936 | 201'116'366 | 96.27 | 104'043'355 | 51.73 | 78'336'866 | 75.29 | 98.07 | 24'398 |
|  | TON_078_003 | Father | V4 | HiSeq | 217'486'676 | 209'413'142 | 96.29 | 193'005'790 | 92.17 | 139'350'233 | 72.20 | 98.10 | 24'756 |
| **25** | TRO_409_001 | Proband | V4 | HiSeq | 253'289'030 | 245'183'378 | 96.80 | 187'698'272 | 76.55 | 141'465'018 | 75.37 | 98.69 | 24'789 |
|  | TRO_409_002 | Mother | V4 | HiSeq | 260'227'436 | 252'257'638 | 96.94 | 203'841'989 | 80.81 | 153'168'646 | 75.14 | 98.70 | 24'884 |
|  | TRO_409_003 | Father | V4 | HiSeq | 270'452'342 | 261'346'120 | 96.63 | 207'918'341 | 79.56 | 158'209'151 | 76.09 | 98.62 | 24'735 |
| **26** | SZP_trio26.P | Proband | V4 | HiSeq | 244'274'446 | 239'066'580 | 97.87 | 182'426'861 | 76.31 | 129'281'130 | 70.87 | 95.47 | 24'267 |
|  | SZP_trio26.M | Mother | V4 | HiSeq | 237'520'124 | 230'167'410 | 96.90 | 185'216'129 | 80.47 | 130'301'608 | 70.35 | 95.4 | 24'217 |
|  | SZP_trio26.F | Father | V4 | HiSeq | 231'675'360 | 224'877'378 | 97.07 | 177'288'040 | 78.84 | 125'098'586 | 70.56 | 95.32 | 24'115 |
| **27** | SZP_trio27.P | Proband | V4 | HiSeq | 236'010'504 | 226'648'426 | 96.03 | 182'929'967 | 80.71 | 124'986'786 | 68.33 | 94.98 | 23'689 |
|  | SZP_trio27.M | Mother | V4 | HiSeq | 247'051'424 | 238'343'122 | 96.48 | 172'468'882 | 72.36 | 123'085'676 | 71.37 | 95.85 | 23'691 |
|  | SZP_trio27.F | Father | V4 | HiSeq | 253'704'240 | 244'100'734 | 96.21 | 189'137'924 | 77.48 | 134'531'464 | 71.13 | 95.83 | 24'054 |
| **28** | SZP_trio28.P | Proband | V4 | HiSeq | 547'704'118 | 532'909'934 | 97.30 | 422'148'433 | 79.22 | 329'475'075 | 78.05 | 98.86 | 26'815 |
|  | SZP_trio28.F | Father | V4 | HiSeq | 263'287'180 | 253'770'276 | 96.39 | 189'338'093 | 74.61 | 134'782'805 | 71.19 | 95.82 | 24'022 |
|  | SZP_trio28.M | Mother | V4 | HiSeq | 242'725'490 | 233'900'102 | 96.36 | 181'645'082 | 77.66 | 129'447'071 | 71.26 | 95.79 | 24'010 |
| **29** | SZP_trio29.P | Proband | V4 | HiSeq | 522'321'376 | 506'282'682 | 96.93 | 298'474'726 | 58.95 | 234'333'324 | 78.51 | 98.84 | 27'230 |
|  | SZP_trio29.M | Mother | V4 | HiSeq | 214'636'894 | 207'269'672 | 96.57 | 162'498'232 | 78.40 | 116'293'064 | 71.57 | 95.72 | 24'379 |
|  | SZP_trio29.F | Father | V4 | HiSeq | 269'382'506 | 260'947'140 | 96.87 | 177'051'398 | 67.85 | 125'623'831 | 70.95 | 95.73 | 23'507 |
| **30** | SZP_trio30.P | Proband | V4 | HiSeq | 236'322'078 | 230'934'378 | 97.72 | 168'646'827 | 73.03 | 119'301'095 | 70.74 | 95.43 | 23'952 |
|  | SZP_trio30.M | Mother | V4 | HiSeq | 267'354'182 | 261'664'756 | 97.87 | 183'964'257 | 70.31 | 128'590'979 | 69.90 | 95.46 | 24'013 |
|  | SZP_trio30.F | Father | V4 | HiSeq | 261'706'798 | 255'862'234 | 97.77 | 182'562'438 | 71.35 | 126'127'194 | 69.09 | 95.38 | 24'174 |
| **31** | SZP_trio31.P | Proband | V4 | HiSeq | 239'093'340 | 234'245'348 | 97.97 | 180'485'900 | 77.05 | 129'360'274 | 71.67 | 95.69 | 23'778 |
|  | SZP_trio31.M | Mother | V4 | HiSeq | 517'004'950 | 503'025'738 | 97.30 | 282'772'120 | 56.21 | 207'858'372 | 73.51 | 98.85 | 26'982 |
|  | SZP_trio31.P | Father | V4 | HiSeq | 293'571'968 | 286'223'064 | 97.50 | 206'910'421 | 72.29 | 147'917'555 | 71.49 | 95.85 | 24'309 |
| **32** | SP-197-001 | Proband | V4 | HiSeq | 175'668'290 | 169'295'782 | 96.37 | 135'595'190 | 80.09 | 103'345'489 | 76.22 | 98.44 | 24'583 |
|  | SP-197-002 | Father | V4 | HiSeq | 179'918'764 | 173'203'564 | 96.27 | 125'027'486 | 72.19 | 95'077'333 | 76.05 | 98.48 | 26'488 |
|  | SP-197-003 | Mother | V4 | HiSeq | 219'941'018 | 212'850'282 | 96.78 | 175'872'591 | 82.63 | 136'290'660 | 77.49 | 98.49 | 26'974 |
| **33** | SP-198-001 | Proband | V4 | HiSeq | 223'204'376 | 215'556'852 | 96.57 | 168'835'679 | 78.33 | 130'318'421 | 77.19 | 98.58 | 24'719 |
|  | SP-198-002 | Father | V4 | HiSeq | 195'309'328 | 188'440'142 | 96.48 | 143'123'325 | 75.95 | 109'563'548 | 76.55 | 98.54 | 24'489 |
|  | SP-198-003 | Mother | V4 | HiSeq | 205'838'794 | 198'559'962 | 96.95 | 148'383'410 | 74.36 | 115'747'876 | 78.01 | 98.71 | 28'692 |
| **34** | SP-226-001 | Proband | V4 | HiSeq | 231'314'652 | 222'987'586 | 96.40 | 190'571'640 | 85.46 | 149'496'785 | 78.45 | 98.42 | 26'328 |
|  | SP-226-002 | Father | V4 | HiSeq | 226'157'982 | 217'452'528 | 96.15 | 186'881'238 | 85.94 | 144'398'893 | 77.27 | 98.58 | 24'288 |
|  | SP-226-003 | Mother | V4 | HiSeq | 208'115'176 | 201'473'184 | 96.81 | 133'282'612 | 66.15 | 106'603'532 | 79.98 | 98.39 | 24'162 |
| **35** | SP-227-001 | Proband | V4 | HiSeq | 213'972'710 | 206'713'864 | 96.61 | 137'848'463 | 66.69 | 110'715'016 | 80.32 | 98.38 | 24'147 |
|  | SP-227-002 | Father | V4 | HiSeq | 244'115'214 | 235'950'564 | 96.66 | 163'085'497 | 69.12 | 131'022'381 | 80.34 | 98.45 | 25'900 |
|  | SP-227-003 | Mother | V4 | HiSeq | 280'920'628 | 271'841'122 | 96.77 | 160'021'127 | 58.87 | 127'906'346 | 79.93 | 98.43 | 24'354 |
| **36** | SP-234-001 | Proband | V4 | HiSeq | 214'705'850 | 208'023'226 | 96.89 | 147'447'482 | 70.88 | 119'207'155 | 80.85 | 98.32 | 26'333 |
|  | SP-234-002 | Father | V4 | HiSeq | 189'415'032 | 169'800'094 | 89.64 | 134'123'137 | 78.98 | 106'005'214 | 79.04 | 98.48 | 24'168 |
|  | SP-234-003 | Mother | V4 | HiSeq | 211'585'358 | 190'605'584 | 90.08 | 151'906'505 | 79.70 | 120'612'477 | 79.40 | 98.30 | 26'421 |
| **37** | SP-236-001 | Proband | V4 | HiSeq | 186'585'030 | 180'483'678 | 96.73 | 114'984'571 | 63.71 | 92'374'870 | 80.34 | 98.64 | 24'790 |
|  | SP-236-002 | Father | V4 | HiSeq | 261'209'790 | 252'068'554 | 96.50 | 150'054'135 | 59.53 | 120'892'978 | 80.57 | 98.56 | 24'873 |
|  | SP-236-003 | Mother | V4 | HiSeq | 210'158'612 | 202'683'186 | 96.44 | 137'281'600 | 67.73 | 105'459'845 | 76.82 | 98.53 | 26'644 |
| **38** | SP-240-001 | Proband | V4 | HiSeq | 230'798'028 | 221'380'932 | 95.92 | 159'648'605 | 72.11 | 122'285'468 | 76.60 | 98.47 | 27'040 |
|  | SP-240-002 | Father | V4 | HiSeq | 197'371'120 | 189'462'372 | 95.98 | 131'630'529 | 69.48 | 100'527'734 | 76.37 | 98.64 | 27'145 |
|  | SP-240-003 | Mother | V4 | HiSeq | 190'598'706 | 183'508'286 | 96.28 | 132'126'635 | 72.00 | 101'680'273 | 76.96 | 98.45 | 27'137 |
| **39** | SP-245-001 | Proband | V4 | HiSeq | 181'978'816 | 175'216'820 | 96.28 | 127'577'721 | 72.81 | 98'347'025 | 77.09 | 98.63 | 26'807 |
|  | SP-245-002 | Father | V4 | HiSeq | 204'066'792 | 196'398'020 | 96.24 | 144'249'465 | 73.45 | 110'162'707 | 76.37 | 98.68 | 24'927 |
|  | SP-245-003 | Mother | V4 | HiSeq | 201'780'394 | 194'217'088 | 96.25 | 128'540'448 | 66.18 | 96'571'577 | 75.13 | 98.56 | 24'897 |
| **40** | 3GF-p | Proband | V4 | HiSeq | 120'584'556 | 115'839'882 | 96.07 | 94'655'430 | 81.71 | 70'795'949 | 74.79 | 98.87 | 24'259 |
|  | 1GF-f | Father | V4 | HiSeq | 120'060'796 | 115'758'730 | 96.42 | 103'859'030 | 89.72 | 77'883'670 | 74.98 | 98.08 | 24'020 |
|  | 2GF-m | Mother | V4 | HiSeq | 119'011'463 | 113'925'157 | 95.73 | 97'986'981 | 86.01 | 72'216'774 | 73.70 | 98.96 | 23'916 |
| **41** | 6TP-p | Proband | V4 | HiSeq | 120'585'321 | 115'342'221 | 95.65 | 94'955'243 | 82.32 | 71'482'671 | 75.28 | 98.95 | 24'029 |
|  | 4TP-f | Father | V4 | HiSeq | 252'528'702 | 241'138'504 | 95.49 | 174'546'849 | 72.38 | 129'916'727 | 74.43 | 98.58 | 24'798 |
|  | 5TP-m | Mother | V4 | HiSeq | 292'360'506 | 279'298'278 | 95.53 | 210'067'787 | 75.21 | 155'840'029 | 74.19 | 98.56 | 25'414 |
| **42** | 9GP-p | Proband | V4 | HiSeq | 258'221'764 | 251'413'020 | 97.36 | 187'554'572 | 74.60 | 143'018'831 | 76.25 | 98.55 | 25'287 |
|  | 7GP-f | Father | V4 | HiSeq | 118'488'061 | 112'396'989 | 94.86 | 105'097'029 | 93.51 | 78'339'506 | 74.54 | 98.98 | 23'781 |
|  | 8GP-m | Mother | V4 | HiSeq | 282'153'854 | 269'206'894 | 95.41 | 183'919'852 | 68.32 | 125'128'367 | 68.03 | 98.51 | 25'040 |
| **43** | 12JC-p | Proband | V4 | HiSeq | 279'134'016 | 271'427'392 | 97.24 | 198'614'092 | 73.54 | 144'586'522 | 72.43 | 98.67 | 24'806 |
|  | 10JC-f | Father | V4 | HiSeq | 122'681'406 | 119'518'346 | 97.42 | 96'941'006 | 81.11 | 73'609'986 | 75.93 | 98.09 | 24'049 |
|  | 11JC-m | Mother | V4 | HiSeq | 152'567'127 | 149'092'433 | 97.72 | 118'509'895 | 79.49 | 89'660'183 | 75.66 | 98.21 | 24'081 |
| **44** | 15LE-p | Proband | V4 | HiSeq | 223'457'156 | 217'847'256 | 97.49 | 174'032'252 | 79.89 | 132'008'662 | 75.85 | 98.50 | 24'537 |
|  | 13LE-f | Father | V4 | HiSeq | 250'609'339 | 244'193'305 | 97.44 | 194'746'875 | 79.75 | 146'737'835 | 75.35 | 98.61 | 24'923 |
|  | 14LE-m | Mother | V4 | HiSeq | 125'303'426 | 122'327'456 | 97.62 | 105'798'173 | 86.49 | 77'108'291 | 72.88 | 98.92 | 23'704 |
| **45** | 18 (3) Patient | Proband | V4 | HiSeq | 197'924'432 | 191'118'166 | 96.56 | 179'425'804 | 93.88 | 136'334'902 | 75.98 | 98.58 | 24'697 |
|  | 16 (1) Father | Father | V4 | HiSeq | 200'984'650 | 190'608'448 | 94.83 | 175'309'563 | 91.97 | 132'818'655 | 75.76 | 98.49 | 30'573 |
|  | 17 (2) Mother | Mother | V4 | HiSeq | 198'037'820 | 190'531'958 | 95.73 | 173'096'797 | 90.85 | 132'924'413 | 76.79 | 98.56 | 24'669 |
| **46** | 88060_1001 | Proband | V4 | HiSeq | 287'941'958 | 281'447'902 | 97.74 | 130'609'066 | 46.41 | 104'007'227 | 79.63 | 98.52 | 24'961 |
|  | 88060_2046 | Father | V4 | HiSeq | 274'855'430 | 267'845'244 | 97.45 | 125'930'913 | 47.02 | 98'803'113 | 78.46 | 98.49 | 25'422 |
|  | 88060_2096 | Mother | V4 | HiSeq | 284'527'334 | 277'367'238 | 97.48 | 147'389'590 | 53.14 | 114'738'119 | 77.85 | 98.52 | 25'641 |
| **47** | 88185_1001 | Proband | V4 | HiSeq | 303'283'194 | 293'041'394 | 96.62 | 129'333'574 | 44.13 | 102'425'231 | 79.19 | 98.49 | 25'551 |
|  | 88185_2046 | Father | V4 | HiSeq | 245'124'622 | 237'212'278 | 96.77 | 122'864'175 | 51.80 | 95'818'758 | 77.98 | 98.43 | 25'315 |
|  | 88185_2096 | Mother | V4 | HiSeq | 177'429'920 | 172'134'566 | 97.02 | 104'036'223 | 60.44 | 82'192'049 | 79.00 | 98.21 | 24'735 |
| **48** | 88536_1001 | Proband | V4 | HiSeq | 223'439'416 | 211'724'198 | 94.76 | 191'638'533 | 90.51 | 149'567'544 | 78.05 | 98.66 | 25'088 |
|  | 88536_2046 | Father | V4 | HiSeq | 223'740'270 | 210'605'650 | 94.13 | 181'349'380 | 86.11 | 142'574'371 | 78.62 | 98.66 | 24'964 |
|  | 88536_2096 | Mother | V4 | HiSeq | 216'228'390 | 205'364'616 | 94.98 | 188'685'912 | 91.88 | 148'205'386 | 78.55 | 98.56 | 25'235 |
| **49** | 98428_1002 | Proband | V4 | HiSeq | 257'517'650 | 248'218'706 | 96.39 | 144'462'580 | 58.20 | 110'573'343 | 76.54 | 98.57 | 24'734 |
|  | 98428_2046 | Father | V4 | HiSeq | 258'860'840 | 248'902'828 | 96.15 | 218'217'157 | 87.67 | 167'191'605 | 76.62 | 98.75 | 25'640 |
|  | 98428_2096 | Mother | V4 | HiSeq | 235'910'578 | 229'188'440 | 97.15 | 201'293'325 | 87.83 | 155'481'724 | 77.24 | 98.62 | 25'076 |
| **50** | 98706_1002 | Proband | V4 | HiSeq | 276'650'464 | 267'565'034 | 96.72 | 235'748'479 | 88.11 | 179'350'802 | 76.08 | 98.78 | 25'092 |
|  | 98706_2046 | Father | V4 | HiSeq | 239'684'304 | 231'919'958 | 96.76 | 202'835'007 | 87.46 | 154'687'006 | 76.26 | 98.75 | 24'982 |
|  | 98706_2096 | Mother | V4 | HiSeq | 234'032'514 | 226'921'076 | 96.96 | 132'585'323 | 58.43 | 102'772'949 | 77.51 | 98.44 | 24'876 |
| **51** | 98708_1032 | Proband | V4 | HiSeq | 235'479'864 | 227'288'432 | 96.52 | 193'406'211 | 85.09 | 147'002'359 | 76.01 | 98.64 | 25'164 |
|  | 98708_2046 | Father | V4 | HiSeq | 237'590'902 | 230'708'572 | 97.10 | 208'505'819 | 90.38 | 156'313'671 | 74.97 | 98.70 | 24'732 |
|  | 98708_2096 | Mother | V4 | HiSeq | 266'573'486 | 258'982'044 | 97.16 | 222'123'244 | 85.76 | 171'610'225 | 77.26 | 98.65 | 25'013 |
| **52** | 98757_1001 | Proband | V4 | HiSeq | 271'676'374 | 263'532'296 | 97.00 | 229'051'903 | 86.92 | 176'470'047 | 77.04 | 98.73 | 25'403 |
|  | 98757_2046 | Father | V4 | HiSeq | 230'240'934 | 224'139'638 | 97.35 | 170'395'651 | 76.02 | 130'193'927 | 76.41 | 98.62 | 25'066 |
|  | 98757_2096 | Mother | V4 | HiSeq | 259'171'744 | 252'487'588 | 97.42 | 222'193'960 | 88.00 | 169'160'629 | 76.13 | 98.63 | 25'459 |
| **53** | 98768_1002 | Proband | V4 | HiSeq | 256'057'384 | 248'859'440 | 97.19 | 217'601'989 | 87.44 | 164'252'644 | 75.48 | 98.69 | 25'296 |
|  | 98768_2046 | Father | V4 | HiSeq | 219'217'346 | 213'564'334 | 97.42 | 180'392'755 | 84.47 | 140'297'320 | 77.77 | 98.59 | 24'850 |
|  | 98768_2096 | Mother | V4 | HiSeq | 201'002'192 | 196'461'182 | 97.74 | 171'063'445 | 87.07 | 132'073'150 | 77.21 | 98.55 | 24'827 |
