# Supplementary material for: Exome Sequencing in 53 Sporadic Cases of Schizophrenia Identifies 18 Putative Candidate Genes
Source: PLoS One. 2014 Nov 24;9(11):e112745. doi: 10.1371/journal.pone.0112745 (PMC4242613; doi:10.1371/journal.pone.0112745)
Supplement: Table S3 — Human Splice Finder (HSF) prediction scores for conserved splice site mutations. (DOCX) [file pone.0112745.s006.docx]

**Table S3**: **Human Splice Finder (HSF) prediction scores for conserved splice site mutations**. Variation between wild-type and mutant greater than 10% are considered as damaging

| **EIF3B variant: c.2029-1G>C** |  |  |  |  |  |
| --- | --- | --- | --- | --- | --- |
| ***HSF Matrices*** |  |  |  |  |  |
| **Splice site type** | **Motif** | **New splice site** | **Wild Type** | **Mutant** | **Variation (%)** |
| Acceptor | gccctggccta**g**GT | gccctggccta**c**GT | 77.81 | 48.86 | -37.2 |
|  |  |  |  |  |  |
| ***MaxEnt*** |  |  |  |  |  |
|  | **Ref Motif** | **Ref Score** | **Mut Motif** | **Mut Score** | **Variation (%)** |
|  | gtgtccctgccctggccta**g**GTG | 7.63 | gtgtccctgccctggccta**c**GTG | -0.43 | -105.64 |
|  |  |  |  |  |  |
|  | | | | | |
| **SETD1A variant c.4582-1-2 ag>-** |  |  |  |  |  |
| ***HSF Matrices*** |  |  |  |  |  |
| **Splice site type** | **Motif** | **New splice site** | **Wild Type** | **Mutant** | **Variation (%)** |
| Acceptor | tgtgtctcac**ag**GG | tgtgtctcacggGA | 89.93 | 60.81 | -32.38 |
|  |  |  |  |  |  |
| ***MaxEnt*** |  |  |  |  |  |
|  | **Ref Motif** | **Ref Score** | **Mut Motif** | **Mut Score** | **Variation (%)** |
|  | ctccctgccgtgtgtctcac**ag**G | 0.74 | ctccctgccgtgtgtctcacGGG | 1.51 | 104.05 |
|  | ccctgccgtgtgtctcac**ag**GGG | 10.64 | ccctgccgtgtgtctcacggGAC | 3.37 | -68.33 |
