# Supplementary material for: Exome Sequencing in 53 Sporadic Cases of Schizophrenia Identifies 18 Putative Candidate Genes
Source: PLoS One. 2014 Nov 24;9(11):e112745. doi: 10.1371/journal.pone.0112745 (PMC4242613; doi:10.1371/journal.pone.0112745)
Supplement: Table S4 — List of genes hit at least twice by likely damaging DNVs across all five studies (Girard et al. 2011, Xu et al. 20122, 2012, Gulsuner et al. 2012, Fromer et al. 2014, and this study). (DOCX) [file pone.0112745.s007.docx]

**Table S4**: **List of genes hit at least twice by damaging DNVs across all five studies** (Girard et al. 2011, Xu et al. 2012, Gulsuner et al. 2012, Fromer et al. 2014 and this study).

| **Study** | **Chr.** | **Mutation** | **Gene** | **AA substitution** | **PP2** | **SIFT** |
| --- | --- | --- | --- | --- | --- | --- |
| Gulsuner_2013 | 22 | Missense | CACNA1I | p.797T>M | 1.00 | 0.00 |
| Gulsuner_2013 | 22 | Missense | CACNA1I | p.1311R>H | 1.00 | 0.00 |
| Fromer_2014 | 5 | Missense | CD14 | p.152V>M | 1.00 | 0.05 |
| Fromer_2014 | 5 | Missense | CD14 | p.27L>M | 0.75 | 0.02 |
| Fromer_2014 | 3 | Missense | CRYBG3 | p.2599E>D |  |  |
| Gulsuner_2013 | 3 | Frameshift | CRYBG3 | c.152delTACTA |  |  |
| Karayiorgou_2012 | 1 | Missense | DPYD | p.539G>R | 1.00 | 0.00 |
| Karayiorgou_2012 | 1 | Nonsense | DPYD | p.621W>* |  |  |
| Fromer_2014 | 11 | Frameshift | HSPA8 | p.488*>* |  |  |
| Fromer_2014 | 11 | Missense | HSPA8 | p.473Q>R | 0.35 | 0.00 |
| Fromer_2014 | 11 | Missense | HSPA8 | p.429T>S | 0.92 | 0.14 |
| Fromer_2014 | X | Missense | HUWE1 | p.4237R>C | 1.00 | 0.01 |
| Fromer_2014 | X | Missense | HUWE1 | p.326A>G | 1.00 | 0.00 |
| Gulsuner_2013 | 4 | Nonsense | KIAA1109 | p.2439Q>* |  |  |
| Karayiorgou_2012 | 4 | Missense | KIAA1109 | p.4950Y>D | 1.00 | 0.14 |
| Fromer_2014 | 11 | Missense | KIF18A | p.188V>I | 0.77 | 0.18 |
| Fromer_2014 | 11 | Missense | KIF18A | p.20P>L | 1.00 | 0.00 |
| Gulsuner_2013 | 6 | Missense | LAMA2 | p.1493R>C | 1.00 | 0.00 |
| Karayiorgou_2012 | 6 | Indel | LAMA2 | p.Ser3050ThrfsX27 |  |  |
| Karayiorgou_2012 | 6 | splice-acceptor_-3 | LAMA2 |  |  |  |
| Fromer_2014 | 1 | Missense | LPHN2 | p.372P>R | 1.00 | 0.00 |
| Fromer_2014 | 1 | Nonsense | LPHN2 | p.803R>* |  |  |
| Fromer_2014 | 10 | Nonsense | MKI67 | p.372R>* |  |  |
| Gulsuner_2013 | 10 | Nonsense | MKI67 | p.857K>* |  |  |
| Fromer_2014 | 22 | Missense | MYH9 | p.1285V>M | 0.54 | 0.04 |
| Gulsuner_2013 | 22 | Splice | MYH9 | c.2838(+1)G>T |  |  |
| Fromer_2014 | 2 | Nonsense | NEB | p.639Y>* |  |  |
| Gulsuner_2013 | 2 | Missense | NEB | p.7908T>M | 1.00 | 0.11 |
| Fromer_2014 | 1 | Missense | NIPAL3 | p.172V>M | 0.99 | 0.10 |
| Fromer_2014 | 1 | Nonsense | NIPAL3 | p.398R>* |  |  |
| Fromer_2014 | 16 | Missense | NLRC5 | p.36L>F | 1.00 | 0.00 |
| Karayiorgou_2012 | 16 | splice-donor_1 | NLRC5 |  |  |  |
| Fromer_2014 | 1 | Frameshift | PIK3C2B |  |  |  |
| Fromer_2014 | 1 | Missense | PIK3C2B | p.31Q>R | 0.98 | 0.18 |
| This study | 4 | Missense | RGS12 | p.702R>L | 1.00 | 0.08 |
| Karayiorgou_2012 | 4 | Missense | RGS12 | p.1120P>L | 0.00 | 0.02 |
| Fromer_2014 | 15 | Missense | RYR3 | p.2205V>M | 0.97 | 0.00 |
| Fromer_2014 | 15 | Missense | RYR3 | p.4730I>T | 1.00 | 0.00 |
| Fromer_2014 | 12 | codon-insertion | SLC4A8 |  |  |  |
| Karayiorgou_2012 | 12 | Missense | SLC4A8 | p.683A>V | 1.00 | 0.04 |
| Fromer_2014 | 17 | Missense | STAC2 | p.3E>K | 0.88 | 0.00 |
| Karayiorgou_2012 | 17 | Missense | STAC2 | p.110L>P | 0.90 | 0.33 |
| Fromer_2014 | 1 | Frameshift | TAF13 |  |  |  |
| Fromer_2014 | 1 | Nonsense | TAF13 | p.80R>* |  |  |
