# Supplementary material for: Exome Sequencing in 53 Sporadic Cases of Schizophrenia Identifies 18 Putative Candidate Genes
Source: PLoS One. 2014 Nov 24;9(11):e112745. doi: 10.1371/journal.pone.0112745 (PMC4242613; doi:10.1371/journal.pone.0112745)
Supplement: Table S5 — List of genes carrying de novo protein-altering mutations as reported by Girard et al. (2011), Xu et al. (2012), Gulsuner et al. (2014 and Fromer et al. (2014) and the present study. (DOCX) [file pone.0112745.s008.docx]

**Table S5**: List of genes carrying de novo protein-altering mutations as reported by Girard et al. (2011), Xu et al. (2012), Gulsuner et al. (2012) and Fromer et al. (2014) and the present study

| **Study** | **Gene** | **Nucleotide change** | **Amino acid substitution** |
| --- | --- | --- | --- |
| Fromer_2014 | ABHD12 | G/A | p.214T>M,p.214T>M |
| Fromer_2014 | ACSL4 | G/A | .,p.529R>C,.,p.570R>C |
| Fromer_2014 | ACSL5 | G/A | .,p.47G>D,.,.,.,. |
| Fromer_2014 | ACTA2 | C/T | p.368G>R,p.368G>R |
| Fromer_2014 | ACTL7B | G/T | p.272L>M |
| Fromer_2014 | ACTN1 | T/C | p.437H>R,p.437H>R,p.437H>R |
| Fromer_2014 | ADAMTS1 | G/A | p.381P>L |
| Fromer_2014 | ADAMTS10 | C/T | p.517E>K |
| Fromer_2014 | ADAMTS3 | C/T | p.929R>H |
| Fromer_2014 | AKD1 | ATAGT/A | .,.,.,p.422*>x |
| Fromer_2014 | AKD1 | T/C | p.1750Y>C |
| Fromer_2014 | AKT1 | C/T | p.370R>H,p.370R>H,p.370R>H |
| Fromer_2014 | AKTIP | A/G | .,p.167W>R,.,p.167W>R |
| Fromer_2014 | ALAS1 | C/T | p.357R>C,p.357R>C |
| Fromer_2014 | ALCAM | A/T | p.489N>I,p.489N>I,p.489N>I |
| Fromer_2014 | ALDH1L2 | T/C | p.817D>G,. |
| Fromer_2014 | ALMS1 | C/T | p.2884R>* |
| Fromer_2014 | ANK1 | G/C | p.597S>R,p.630S>R,p.597S>R,p.597S>R,p.597S>R |
| Fromer_2014 | AOX1 | G/A | p.1006G>E |
| Fromer_2014 | ARHGEF11 | G/A | p.608R>C,p.648R>C |
| Fromer_2014 | ASAP1 | C/A | p.411A>S,p.418A>S |
| Fromer_2014 | ASTL | G/A | p.169A>V |
| Fromer_2014 | ATG12 | GGAGT/G | .,.,.,.,.,.,. |
| Fromer_2014 | BAIAP2 | CCCT/C | .,.,. |
| Fromer_2014 | BCAT1 | GT/G | .,.,.,.,. |
| Fromer_2014 | BRSK1 | G/A | .,p.46G>E |
| Fromer_2014 | BTNL2 | C/T | p.65W>* |
| Fromer_2014 | C14orf28 | T/G | p.92F>V |
| Fromer_2014 | C7orf60 | T/C | p.395Y>C |
| Fromer_2014 | CAPN9 | A/G | p.196M>V,p.196M>V |
| Fromer_2014 | CBX8 | G/A | p.110S>L |
| Fromer_2014 | CCDC47 | A/G | p.295L>P |
| Fromer_2014 | CD14 | C/T | p.152V>M,p.152V>M,p.152V>M,p.152V>M |
| Fromer_2014 | CD163 | T/C | p.454N>D,p.454N>D |
| Fromer_2014 | CELSR3 | C/T | p.1277V>M |
| Fromer_2014 | CEP164 | A/G | p.8I>V,p.8I>V |
| Fromer_2014 | CERK | T/C | .,p.189D>G |
| Fromer_2014 | CHMP2A | C/T | p.103M>I,p.103M>I |
| Fromer_2014 | CHSY1 | T/C | p.655Y>C |
| Fromer_2014 | CIC | A/G | p.264H>R |
| Fromer_2014 | CLCN1 | GC/G | .,. |
| Fromer_2014 | CLEC16A | G/A | p.233D>N,p.235D>N |
| Fromer_2014 | CLPX | C/T | p.123R>H |
| Fromer_2014 | CNTN3 | T/A | .,. |
| Fromer_2014 | COL15A1 | G/A | p.189R>Q |
| Fromer_2014 | COL6A2 | G/A | p.484G>R,p.484G>R,p.484G>R |
| Fromer_2014 | CR1L | C/G | p.539P>A |
| Fromer_2014 | CRYBG3 | A/C | p.2599E>D |
| Fromer_2014 | CTNNA2 | G/A | p.315A>T,p.315A>T |
| Fromer_2014 | CUL3 | C/G | p.686E>Q,p.758E>Q,p.752E>Q |
| Fromer_2014 | CUZD1 | A/T | p.527Y>*,.,. |
| Fromer_2014 | DAGLB | G/A | p.385R>*,p.514R>* |
| Fromer_2014 | DARC | CT/C | .,. |
| Fromer_2014 | DCST1 | T/C | p.574L>S,p.599L>S,.,. |
| Fromer_2014 | DLG2 | C/T | .,.,.,.,.,.,. |
| Fromer_2014 | DLL4 | G/A | p.577D>N |
| Fromer_2014 | DNAH9 | G/GC | .,. |
| Fromer_2014 | DPEP2 | C/T | p.91D>N |
| Fromer_2014 | DPM1 | T/C | p.229I>V |
| Fromer_2014 | DUOXA1 | T/A | p.73S>C,.,p.73S>C,.,.,p.73S>C |
| Fromer_2014 | DUOXA2 | GC/G | . |
| Fromer_2014 | DUSP15 | G/T | p.4T>K,p.107T>K,p.4T>K |
| Fromer_2014 | DVL1 | G/A | .,p.351R>W |
| Fromer_2014 | EMR1 | A/T | p.240Y>F,p.292Y>F,p.151Y>F,.,p.292Y>F |
| Fromer_2014 | EPB41L1 | G/A | p.634E>K,.,p.560E>K,p.634E>K,p.560E>K |
| Fromer_2014 | EPHA2 | CG/C | .,. |
| Fromer_2014 | EPYC | G/A | p.188R>* |
| Fromer_2014 | ERF | C/T | p.487R>H |
| Fromer_2014 | ESR2 | G/A | p.197R>W,p.197R>W,p.197R>W,p.197R>W,p.197R>W,p.197R>W,.,.,. |
| Fromer_2014 | EVI5L | G/A | p.182V>I,p.182V>I |
| Fromer_2014 | FAM160B1 | G/A | p.128V>M,p.128V>M |
| Fromer_2014 | FAT4 | A/T | p.859Q>H |
| Fromer_2014 | FBXL19 | G/A | p.590R>H,p.547R>H,. |
| Fromer_2014 | FKBPL | C/G | p.124C>S |
| Fromer_2014 | FN1 | C/T | p.1150V>I,p.1150V>I,p.1150V>I,p.1150V>I,p.1150V>I |
| Fromer_2014 | FN3KRP | A/G | p.65T>A,. |
| Fromer_2014 | GFOD1 | G/A | p.5A>V,p.5A>V,p.108A>V |
| Fromer_2014 | GNB2 | A/C | p.247D>A |
| Fromer_2014 | GRID2 | C/G | p.657T>S |
| Fromer_2014 | GRIN2A | A/G | p.1295I>T,p.1295I>T,. |
| Fromer_2014 | GTF3C2 | G/A | p.595R>W,p.595R>W |
| Fromer_2014 | GTPBP3 | C/CGG | .,.,.,. |
| Fromer_2014 | GTPBP5 | C/T | p.371S>L |
| Fromer_2014 | HIVEP3 | G/A | p.934Q>*,p.934Q>* |
| Fromer_2014 | HSP90AA1 | C/CCT | .,. |
| Fromer_2014 | HSPA8 | A/ATGCC | p.488*>*,.,. |
| Fromer_2014 | HTR2A | A/C | p.208M>R,p.124M>R |
| Fromer_2014 | HUWE1 | G/A | p.4237R>C |
| Fromer_2014 | ILVBL | C/T | p.322R>Q |
| Fromer_2014 | INO80 | C/T | p.277R>H |
| Fromer_2014 | INSRR | G/A | .,p.36R>C |
| Fromer_2014 | INTS6 | T/G | p.255I>L,p.268I>L |
| Fromer_2014 | ITM2C | C/T | p.158T>M,p.168T>M,p.205T>M |
| Fromer_2014 | ITSN1 | C/T | p.368R>*,p.368R>* |
| Fromer_2014 | JARID2 | G/A | p.597G>S,p.769G>S |
| Fromer_2014 | KIAA1244 | C/T | p.480R>C |
| Fromer_2014 | KIAA1244 | A/G | p.1875K>R |
| Fromer_2014 | KIAA1429 | AG/A | .,. |
| Fromer_2014 | KIAA2018 | C/G | p.1439Q>H |
| Fromer_2014 | KIF13B | T/C | p.336N>S |
| Fromer_2014 | KIF18A | C/T | p.188V>I |
| Fromer_2014 | KLHDC1 | ACT/A | . |
| Fromer_2014 | KLHL17 | CCT/C | . |
| Fromer_2014 | KLHL20 | C/T | p.226R>C |
| Fromer_2014 | KRT15 | A/T | .,p.167I>N |
| Fromer_2014 | KRT20 | G/A | p.222T>I |
| Fromer_2014 | LAMA4 | C/T | p.1469A>T,p.1462A>T,p.1462A>T |
| Fromer_2014 | LHX6 | C/T | p.146G>R,p.128G>R,.,p.157G>R,p.157G>R |
| Fromer_2014 | LPAR3 | A/G | p.300M>T |
| Fromer_2014 | LPHN2 | C/G | p.372P>R |
| Fromer_2014 | LRP4 | C/T | p.907G>R |
| Fromer_2014 | MAPK8 | G/A | p.171G>S,p.171G>S,p.171G>S,p.171G>S |
| Fromer_2014 | MARK4 | G/A | .,.,.,. |
| Fromer_2014 | MDM1 | G/A | p.318T>M,p.353T>M |
| Fromer_2014 | MGME1 | A/T | p.253K>* |
| Fromer_2014 | MIF | G/GT | .,. |
| Fromer_2014 | MKI67 | G/A | p.372R>*,p.732R>* |
| Fromer_2014 | MOV10 | A/AC | .,. |
| Fromer_2014 | MPG | C/T | p.207R>C,p.195R>C,p.212R>C |
| Fromer_2014 | MUC6 | G/C | p.1049L>V |
| Fromer_2014 | MYADML2 | A/G | p.203L>P |
| Fromer_2014 | MYH11 | C/T | p.1137E>K,p.1137E>K,p.1130E>K,p.1130E>K |
| Fromer_2014 | MYH7B | G/A | .,. |
| Fromer_2014 | MYH9 | C/T | p.1285V>M |
| Fromer_2014 | MYO18A | C/T | p.1219R>H,p.1219R>H |
| Fromer_2014 | MYO18B | C/T | p.1535R>C |
| Fromer_2014 | MYOF | G/A | p.1371P>S,p.1358P>S |
| Fromer_2014 | MYOZ2 | G/T | p.146K>N |
| Fromer_2014 | NALCN | G/A | p.1264S>L |
| Fromer_2014 | NAP1L2 | CT/C | . |
| Fromer_2014 | NCKIPSD | GGTGGGATCTGGGAAGATGGAA/G | .,.,. |
| Fromer_2014 | NEB | A/T | p.639Y>*,p.639Y>*,p.639Y>*,p.639Y>* |
| Fromer_2014 | NFASC | T/C | p.408C>R,p.408C>R,p.419C>R,p.419C>R,p.402C>R,p.419C>R |
| Fromer_2014 | NIPAL3 | G/A | p.172V>M |
| Fromer_2014 | NIPAL3 | C/T | p.398R>* |
| Fromer_2014 | NIPBL | C/G | .,p.2773Y>* |
| Fromer_2014 | NLRC5 | C/T | p.36L>F |
| Fromer_2014 | NLRP1 | G/T | .,p.1380S>R,p.1424S>R,p.1394S>R,p.1350S>R |
| Fromer_2014 | NOS3 | T/G | p.941S>A |
| Fromer_2014 | NR0B2 | G/A | p.86R>W |
| Fromer_2014 | NR2E3 | TCCCCCGG/T | .,. |
| Fromer_2014 | NRXN1 | T/C | p.888E>G,p.848E>G |
| Fromer_2014 | NVL | G/A | p.233R>C,p.331R>C,p.422R>C,p.316R>C |
| Fromer_2014 | PAQR8 | G/A | p.54G>S |
| Fromer_2014 | PAQR9 | C/T | p.47W>* |
| Fromer_2014 | PAX5 | G/A | p.207P>L |
| Fromer_2014 | PCDH10 | C/A | p.201L>M,p.201L>M |
| Fromer_2014 | PCDHAC2 | G/T | .,p.50E>*,.,.,.,.,.,.,.,.,.,.,.,.,.,.,.,.,p.50E>* |
| Fromer_2014 | PCNT | C/G | p.1855R>G |
| Fromer_2014 | PDZD2 | C/A | p.1137P>H |
| Fromer_2014 | PHF7 | C/T | .,p.31P>S,.,p.31P>S |
| Fromer_2014 | PHLDB1 | G/A | p.565G>R,p.565G>R,p.565G>R |
| Fromer_2014 | PI15 | C/T | p.85A>V |
| Fromer_2014 | PIK3C2B | ACTTACCG/A | .,. |
| Fromer_2014 | POGZ | CA/C | .,.,.,.,. |
| Fromer_2014 | POM121C | G/A | p.751T>M |
| Fromer_2014 | POMT1 | C/T | p.388R>C,p.334R>C,p.388R>C,p.271R>C,p.410R>C |
| Fromer_2014 | PRRC2A | TC/T | .,p.2158*>x,.,p.2158*>x |
| Fromer_2014 | PRSS38 | C/T | p.272P>S |
| Fromer_2014 | PRUNE2 | A/C | p.519D>E |
| Fromer_2014 | PSAP | T/C | p.1M>V,p.1M>V,p.1M>V,p.1M>V,p.1M>V,p.1M>V |
| Fromer_2014 | PSPC1 | T/C | p.65I>V,.,. |
| Fromer_2014 | PTK2B | A/G | .,p.225K>E,.,p.225K>E,.,p.225K>E,.,p.225K>E |
| Fromer_2014 | PTPRF | T/C | p.1585S>P,p.1576S>P |
| Fromer_2014 | PTPRG | C/G | .,p.1105T>S,.,.,. |
| Fromer_2014 | PTPRJ | C/T | p.300R>W,p.300R>W |
| Fromer_2014 | PUF60 | C/T | p.448G>R,p.473G>R,p.445G>R,p.490G>R,p.462G>R,p.431G>R,p.474G>R,p.491G>R |
| Fromer_2014 | PYCARD | A/C | p.126V>G,p.107V>G |
| Fromer_2014 | RAD54L2 | G/A | p.440R>Q |
| Fromer_2014 | RASSF5 | CA/C | .,.,p.337*>x,. |
| Fromer_2014 | RELN | T/C | p.1981Y>C,p.1981Y>C |
| Fromer_2014 | RYR3 | G/A | p.2205V>M,p.2205V>M |
| Fromer_2014 | SAFB2 | C/A | p.580E>D |
| Fromer_2014 | SCN2A | A/G | .,.,.,.,.,. |
| Fromer_2014 | SCRN1 | G/T | p.300P>T,p.320P>T,p.232P>T,p.300P>T |
| Fromer_2014 | SGSM2 | C/T | p.913R>C,p.958R>C |
| Fromer_2014 | SH3TC2 | A/T | p.575L>M |
| Fromer_2014 | SHANK1 | T/TG | . |
| Fromer_2014 | SHKBP1 | C/G | p.553S>C |
| Fromer_2014 | SHOX2 | T/C | p.299K>E,p.335K>E,p.311K>E |
| Fromer_2014 | SHROOM1 | A/G | p.560L>P,p.560L>P |
| Fromer_2014 | SLC4A8 | C/CCGGAGA | .,.,.,.,. |
| Fromer_2014 | SMARCC2 | CTT/C | .,.,. |
| Fromer_2014 | SMTNL1 | C/T | p.340R>W |
| Fromer_2014 | SND1 | A/G | p.337D>G |
| Fromer_2014 | SORBS2 | C/A | .,.,.,.,p.621Q>H,.,p.817Q>H,.,p.717Q>H |
| Fromer_2014 | SQLE | C/G | p.470A>G |
| Fromer_2014 | SSH2 | C/T | p.493E>K |
| Fromer_2014 | SSPO | G/T | p.1686G>C |
| Fromer_2014 | STAC2 | C/T | p.3E>K |
| Fromer_2014 | STK10 | A/G | p.522L>P |
| Fromer_2014 | STK38L | A/C | p.182K>N |
| Fromer_2014 | SULF2 | C/T | p.517G>R,p.517G>R,p.517G>R |
| Fromer_2014 | SYNE1 | T/G | p.7077E>A,p.7148E>A |
| Fromer_2014 | TAF13 | CTCTT/C | . |
| Fromer_2014 | TANC1 | G/A | p.1113R>H,p.1121R>H |
| Fromer_2014 | TANC2 | C/T | p.794A>V |
| Fromer_2014 | TBC1D10B | C/T | p.491G>R |
| Fromer_2014 | TBXA2R | C/G | p.123G>A,p.123G>A |
| Fromer_2014 | TCP11L2 | T/C | p.73S>P |
| Fromer_2014 | TGM2 | G/A | p.556T>M |
| Fromer_2014 | THRAP3 | G/A | p.270V>M |
| Fromer_2014 | TINF2 | A/C | p.394L>V,. |
| Fromer_2014 | TLK1 | GT/G | .,.,. |
| Fromer_2014 | TLR5 | G/A | p.850P>L |
| Fromer_2014 | TM9SF3 | G/A | p.557A>V |
| Fromer_2014 | TMEM201 | CCT/C | .,. |
| Fromer_2014 | TMEM87B | T/C | p.230Y>H |
| Fromer_2014 | TNRC18 | C/T | p.2788W>* |
| Fromer_2014 | TNRC6C | C/G | p.1223L>V,p.1226L>V |
| Fromer_2014 | TP53I13 | C/T | p.166R>* |
| Fromer_2014 | TPR | T/C | p.1720T>A,. |
| Fromer_2014 | TRH | C/T | p.119R>* |
| Fromer_2014 | TSHZ2 | G/A | p.970R>Q,p.973R>Q |
| Fromer_2014 | TSKS | G/A | p.2A>V |
| Fromer_2014 | TSPYL2 | G/A | p.289R>H |
| Fromer_2014 | TTC28 | C/A | p.1108E>* |
| Fromer_2014 | TTN | C/T | .,p.2354R>H,.,p.2354R>H,.,p.2308R>H,.,p.2354R>H,.,p.2354R>H,.,p.2308R>H,.,p.2308R>H |
| Fromer_2014 | UFL1 | A/T | p.446K>* |
| Fromer_2014 | VPS13C | GAT/G | .,.,.,. |
| Fromer_2014 | WARS | G/A | p.196T>M,p.196T>M,p.155T>M,p.155T>M |
| Fromer_2014 | WIZ | G/A | p.382R>C |
| Fromer_2014 | WRN | C/T | p.1146S>L |
| Fromer_2014 | XKR9 | G/A | p.87G>R |
| Fromer_2014 | XPOT | CCT/C | .,p.963*>x |
| Fromer_2014 | YARS | A/G | p.104M>T |
| Fromer_2014 | YTHDC1 | C/CAT | .,. |
| Fromer_2014 | ZBTB45 | C/T | p.269G>R |
| Fromer_2014 | ZC3H10 | C/T | p.428R>C |
| Fromer_2014 | ZC3H6 | G/C | p.382R>T |
| Fromer_2014 | ZDHHC5 | G/T | p.648E>* |
| Fromer_2014 | ZEB1 | C/T | p.936R>*,p.932R>*,p.935R>*,p.885R>*,p.953R>*,p.952R>* |
| Fromer_2014 | ZMYND11 | TCAGA/T | .,.,.,.,.,.,. |
| Fromer_2014 | ZNF326 | G/C | p.12C>S,p.12C>S |
| Fromer_2014 | ZNF407 | A/AG | . |
| Fromer_2014 | ZNF445 | G/A | p.49P>L |
| Fromer_2014 | ZNF536 | G/T | p.380R>L |
| Fromer_2014 | ZNF607 | C/A | p.617E>*,p.618E>* |
| Fromer_2014 | ZNF77 | G/A | p.257R>W |
| Guipponi | AHDC1 | c.C1459T | p.R487W |
| Guipponi | C5orf4 | c.G893A | p.G298E |
| Guipponi | C9orf172 | c.G2855T | p.C952F |
| Guipponi | CHD5 | c.G3943A | p.V1315M |
| Guipponi | CHRNG | c.C511T | p.Q171X |
| Guipponi | DDX20 | c.G163C | p.D55H |
| Guipponi | EIF3B | c.2029-1G>C |  |
| Guipponi | FAM189A2 | c.C478T | p.P160S |
| Guipponi | KDM3B | c.C4216T | p.R1406W |
| Guipponi | KIAA0430 | c.A340C | p.M114L |
| Guipponi | KY | c.C851A | p.T284N |
| Guipponi | LRRC4 | c.C327G | p.N109K |
| Guipponi | MAP4K4 | c.C1795T | p.R599X |
| Guipponi | QSER1 | c.T4100C | p.I1367T |
| Guipponi | RGS12 | c.G2105T | p.R702L |
| Guipponi | RSBN1 | c.A1337C | p.K446T |
| Guipponi | SETD1A | c.4582delAG> - |  |
| Guipponi | SHARPIN | c.T427C | p.S143P |
| Guipponi | SLC22A23 | c.T734G | p.L245R |
| Guipponi | TMEM8C | c.C184T | p.R62C |
| Guipponi | TNNI1 | c.A516G | p.G173S |
| Guipponi | USP10 | c.C2197T | p.R733W |
| Gulsuner_2013 | ADAMTS17 | T/C | R960G |
| Gulsuner_2013 | ADCY9 | T/C | S784G |
| Gulsuner_2013 | ASAP2 | G/A | V596M |
| Gulsuner_2013 | BLNK | G/A | R49stop |
| Gulsuner_2013 | BSND | G/A | E88K |
| Gulsuner_2013 | CACNA1I | C/T | T797M |
| Gulsuner_2013 | CELSR2 | C/G | A423G |
| Gulsuner_2013 | CHEK2 | T/C | N229S |
| Gulsuner_2013 | CLTCL1 | C/G | V641L |
| Gulsuner_2013 | DCDC5 | G/C | P27R |
| Gulsuner_2013 | DNMT1 | G/A | P783T |
| Gulsuner_2013 | DTX1 | C/G | L69V |
| Gulsuner_2013 | FILIP1 | G/A | R1119W |
| Gulsuner_2013 | FLAD1 | /ins 1 | c.476insT |
| Gulsuner_2013 | FNDC9 | C/G | A1269P |
| Gulsuner_2013 | FRY | A/G | T1538A |
| Gulsuner_2013 | GLIPR1L2 | G/A | W80stop |
| Gulsuner_2013 | GLS | T/G | L606R |
| Gulsuner_2013 | HIF1A | C/G | L386V |
| Gulsuner_2013 | HIVEP1 | G/T | C259F |
| Gulsuner_2013 | ITGA3 | C/G | P680A |
| Gulsuner_2013 | KCNU1 | G/T | E841stop |
| Gulsuner_2013 | KIAA1109 | C/T | Q2439stop |
| Gulsuner_2013 | LAMA2 | C/T | R1493C |
| Gulsuner_2013 | LDLRAD4 | G/A | G22S |
| Gulsuner_2013 | MIOS | C/G | A209G |
| Gulsuner_2013 | N4BP2L2 | C/A | V239F |
| Gulsuner_2013 | NCAN | C/T | P1219L |
| Gulsuner_2013 | NCOR2 | G/A | R1954C |
| Gulsuner_2013 | NUP214 | /ins 1 | c.1570insC |
| Gulsuner_2013 | ODZ1 | C/T | W1839stop |
| Gulsuner_2013 | RBBP5 | G/A | H304Y |
| Gulsuner_2013 | RUNX3 | C/T | R211H |
| Gulsuner_2013 | RUSC1 | G/A | R780Q |
| Gulsuner_2013 | SERPINI1 | C/T | R393stop |
| Gulsuner_2013 | SLC18A2 | A/C | c.64A>C |
| Gulsuner_2013 | SLC25A1 | G/A | R285W |
| Gulsuner_2013 | SLIT2 | A/C | N521T |
| Gulsuner_2013 | SLIT3 | C/T | C1011Y |
| Gulsuner_2013 | SNX31 | C/G | L179F |
| Gulsuner_2013 | ST3GAL6 | /ins 1 | c.842insA |
| Gulsuner_2013 | TLK2 | G/A | R303Q |
| Gulsuner_2013 | VPS11 | A/G | T664A |
| Xu_2012 | ADCY7 | c.3058A>G | *p.S1020G* |
| Xu_2012 | ANO9 | c.362G>T | *p.R121L* |
| Xu_2012 | AP2A2 | c.412G>A | *p.V138M* |
| Xu_2012 | BCORL1 | c.1165A>T | *p.I389F* |
| Xu_2012 | BIRC6 | c.9926C>T | *p.S3309F* |
| Xu_2012 | BRPF1 | c.2921-1 | *r.spl?* |
| Xu_2012 | CAMK4 | c.862C>T | *p.R288W* |
| Xu_2012 | CCDC108 | c.314A>G | *p.N105S* |
| Xu_2012 | CCDC39 | c.48_50delCAGinsG | *p.Gln17ValfsX6* |
| Xu_2012 | CCDC84 | c.845T>C | *p.V282A* |
| Xu_2012 | CIT | c.238T>C | *p.Y80H* |
| Xu_2012 | CUGBP2/CELF2 | c.1003+2 | *r.spl?* |
| Xu_2012 | DDHD2 | c.1335_1340delAAACTCinsA | *p.Ser447GlyfsX3* |
| Xu_2012 | DGCR2 | c.1163C>G | *p.P388R* |
| Xu_2012 | DPYD | c.1615G>A | *p.G539R* |
| Xu_2012 | EMR3 | c.1138C>A | *p.L380M* |
| Xu_2012 | ESAM | c.166delGinsCGCTG | *p.Val56ArgfsX48* |
| Xu_2012 | FAM13C | c.713C>T | *p.S238L* |
| Xu_2012 | FAM3D | c.440A>G | *p.Y147C* |
| Xu_2012 | FASTKD5 | c.1238C>G | *p.A413G* |
| Xu_2012 | FCGBP | c.1516G>T | *p.D506Y* |
| Xu_2012 | GNAO1 | c.679G>A | *p.A227T* |
| Xu_2012 | GPR115 | c.1874T>A | *p.L625H* |
| Xu_2012 | GPR153 | c.305C>T | *p.T102I* |
| Xu_2012 | HIST1H1E | c.555_557delCAAinsC | *p.Lys186GlufsX9* |
| Xu_2012 | HMGCR | c.1468C>T | *p.R490C* |
| Xu_2012 | IFT140 | c.2368G>A | *p.E790K* |
| Xu_2012 | KDM5C | c.510_511delGAinsA | *p.Ile171SerfsX8* |
| Xu_2012 | KIAA0467 | c.2998delCinsCCA | *p.Gln1001HisfsX47* |
| Xu_2012 | KLF12 | c.995C>G | *p.S332C* |
| Xu_2012 | LCT | c.5218T>C | *p.Y1740H* |
| Xu_2012 | MACF1 | c.12097C>T | *p.R4033W* |
| Xu_2012 | MBTPS1 | c.1655C>T | *p.S552F* |
| Xu_2012 | NPRL2 | c.691G>A | *p.G231S* |
| Xu_2012 | NUP54 | c.1381C>T | *p.R461** |
| Xu_2012 | OR4C46 | c.604G>A | *p.G202R* |
| Xu_2012 | P2RY2 | c.76C>T | *p.R26C* |
| Xu_2012 | PLCL2 | c.37T>A | *p.C13S* |
| Xu_2012 | PML | c.224C>T | *p.T75M* |
| Xu_2012 | PRDX6 | c.96-2 | *r.spl?* |
| Xu_2012 | PSG2 | c.600G>T | *p.R200S* |
| Xu_2012 | RARG | c.625A>T | *p.K209** |
| Xu_2012 | RASGRP1 | c.1022G>A | *p.R341Q* |
| Xu_2012 | RB1CC1 | c.3682_3684delGAAinsA | *p.Glu1228ThrfsX7* |
| Xu_2012 | RECK | c.206G>A | *p.R69Q* |
| Xu_2012 | RRP1B | c.914A>T | *p.D305V* |
| Xu_2012 | SAP30BP | c.868G>A | *p.G290S* |
| Xu_2012 | SLC26A8 | c.1849G>A | *p.E617K* |
| Xu_2012 | SSBP3 | c.98C>T | *p.A33V* |
| Xu_2012 | STAG1 | c.667A>T | *p.T223S* |
| Xu_2012 | STAP2 | c.66T>A | *p.Y22** |
| Xu_2012 | SYNGAP1 | c.3583-1 | *r.spl?* |
| Xu_2012 | SYNM | c. 3540G>A | *p.A1180T* |
| Xu_2012 | TBC1D14 | c.1397T>C | *p.I466T* |
| Xu_2012 | TRAK1 | c.2033A>G | *p.H678R* |
| Xu_2012 | TREM2 | c.292C>T | *p.R98W* |
| Xu_2012 | TRRAP | c.883A>T | *p.I295F* |
| Xu_2012 | UBQLN1 | c.965A>T | *p.N322I* |
| Xu_2012 | UBR5 | c.5150C>A | *p.A1717D* |
| Xu_2012 | UGT1A3 | c.94T>A | *p.L32M* |
| Xu_2012 | UNC13C | c.3850G>T | *p.V1284F* |
| Xu_2012 | URB2 | c.4000G>T | *p.E1334** |
| Xu_2012 | WDR11 | c.3242G>A | *p.R1081H* |
| Xu_2012 | XPR1 | c.1771delAinsATTGCTTTGTTGCC | *p.Ile592AlafsX13* |
| Xu_2012 | ZBTB40 | c.3313C>T | *p.R1105W* |
| Girard_2011 | *ALS2CL* | G:A | p.Arg733X |
| Girard_2011 | *CCDC137* | A:G | p.Tyr125Cys |
| Girard_2011 | *CHD4* | G:A | p.Arg576Trp |
| Girard_2011 | *KDM2B* | C:T | p.Gly745Ser |
| Girard_2011 | *KPNA1* | C:A | p.Glu448X |
| Girard_2011 | *LRP1* | C:A | p.Tyr2200X |
| Girard_2011 | *NRIP1* | T:G | p.Lys722Thr |
| Girard_2011 | *SBNO1* | G:C | p.Asn886Lys |
| Girard_2011 | *ZNF480* | C:T | p.Arg480X |
