# Supplementary material for: Exome Sequencing in 53 Sporadic Cases of Schizophrenia Identifies 18 Putative Candidate Genes
Source: PLoS One. 2014 Nov 24;9(11):e112745. doi: 10.1371/journal.pone.0112745 (PMC4242613; doi:10.1371/journal.pone.0112745)
Supplement: Text S1 — Detailed sample characteristics. (DOCX) [file pone.0112745.s009.docx]

**Text S1: Detailed sample characteristics**

The schizophrenia trios (case and healthy parents) used in this study were collected at 5 different psychiatric hospitals: the Department of Psychiatry at the University Hospitals of Geneva (Switzerland), the Centre Hospitalier du Rouvray at Sotteville les Rouen (France), the 1^st^ Department of Psychiatry at the Athens University Medical School (Greece), the Pôle de Psychiatrie at the A. Chenevier-H. Mondor Hospital of Créteil (France), the Division of Molecular and Clinical Neurobiology, Department of Psychiatry at the University of Munich (Germany)(the “PAGES” sample), and by the Johns Hopkins Epidemiology Genetics in Psychiatry (USA). This research project was approved by the ethics committee of all participating centers. All participants provided their written informed consent.

The SCZ cohort from Geneva (Switzerland) consisted of 9 affected cases (6 males and 3 females; SZPtrio1 to 9) and their healthy parents. Each patient and their parents were interviewed by trained psychiatrists using the French version of the Diagnostic Interview for Genetic Studies (DIGS) {Nurnberger, 1994 #120} and the Positive and Negative Syndrome Scale (PANSS) {Kay, 1987 #121}. Five of the patients (56%) were diagnosed with non-organic psychosis, 3 (33%) were diagnosed with paranoid schizophrenia and 1 (11%) was diagnosed with simple schizophrenia. The mean age at onset was 18.2 years.

The SCZ sample from Rouen (France) comprised 16 trios (14 males and 2 females; SZPtrio10 to 25; Table S1). Each patient and their parents were interviewed by trained psychiatrists or psychologists using one or two of the following questionnaires: the Diagnostic Interview for Genetic Studies (DIGS) {Nurnberger, 1994 #120}, the Positive and Negative Syndrome Scale (PANSS) {Kay, 1987 #121}, the Mini International Neuropsychiatric Interview (MINI) {Sheehan, 1998 #122}, the Brief Psychiatric Rating Scale (BPRS) {Andersen, 1989 #126} and the Schedule for Affective Disorder and Schizophrenia-Lifetime Anxiety version (SADS-LA) {Mannuzza, 1986 #127}. Twelve of the patients (75%) were diagnosed with paranoid schizophrenia, 2 (12.5%) with disorganized schizophrenia and 2 (12.5%) with schizoaffective disorder. The mean age at onset and the mean age at recruitment were 21.9 and 35.8 years, respectively.

The SCZ cohort from Munich “PAGES” (Germany) consisted of 6 trios (5 male and 1 female patients; SZPtrio26 to 31; Table S1), and their healthy parents. Each patient and their parents were interviewed by trained psychiatrists using the Structured Clinical Interview for DSM-IV (<http://www.scid4.org/info/refscid.html>) and the Positive and Negative Syndrome Scale (PANSS) {Kay, 1987 #121}. Five of the cases (83.3%) were diagnosed with paranoid schizophrenia and 1 (16.7%) was diagnosed with disorganized schizophrenia. The mean age at onset and the mean age at recruitment were 23 and 31.16 years, respectively.

The SCZ sample from Créteil (France) consisted of 8 trios (5 male and 3 female patients; SZPtrio32 to 39; Table S1), and their healthy parents. Each patient and their parents were interviewed by trained psychiatrists using the French version of the Diagnostic Interview for Genetic Studies (DIGS) {Nurnberger, 1994 #120} and the Positive and Negative Syndrome Scale (PANSS) {Kay, 1987 #121}. Seven of the cases (87.5%) were diagnosed with paranoid schizophrenia and one (12.5%) was diagnosed with undifferentiated schizophrenia. The mean age at onset and the mean age at recruitment were 22.6 and 35.3 years, respectively.

The SCZ cohort from Athens (Greece) consisted of 6 trios (4 female and 2 male patients, as well as their parents; SZPtrio40 to 45; Table S1). Each patient and their parents were interviewed by a trained psychiatrist (GG) using the Greek versions of the *Mini* International Neuropsychiatric Interview (MINI) {Sheehan, 1998 #122} and the Positive and Negative Syndrome Scale (PANSS) {Kay, 1987 #121}. Among the patients of the sample, 4 (66.6%) were diagnosed with paranoid schizophrenia, and 2 (33.3%) were diagnosed with disorganized schizophrenia. The mean age at onset and the mean age at recruitment were 21 and 29 years, respectively.

The SCZ sample from Baltimore (USA) consisted of 8 (7 male and 1 female patients, as well as their unaffected parents; SZPtrio46 to 53; Table S1). Each patient and their parents were interviewed by trained psychiatrists using the Diagnostic Interview for Genetic Studies (DIGS) and the Positive and Negative Syndrome Scale (PANSS) {Kay, 1987 #121}. Among the patients of the sample, five (62.5%) were diagnosed with undifferentiated schizophrenia and 3 (37.5%) were diagnosed with schizoaffective disorder. The mean age at onset and the mean age at assessment were 19.5 and 40.1 years, respectively.
